# Mek1 coordinates meiotic progression with DNA break repair by directly phosphorylating and inhibiting the yeast pachytene exit regulator Ndt80

**DOI:** 10.1371/journal.pgen.1007832

**Published:** 2018-11-29

**Authors:** Xiangyu Chen, Robert Gaglione, Trevor Leong, Lauren Bednor, Teresa de los Santos, Ed Luk, Michael Airola, Nancy M. Hollingsworth

**Affiliations:** Department of Biochemistry and Cell Biology, Stony Brook University, Stony Brook, New York, United States of America; National Cancer Institute, UNITED STATES

## Abstract

Meiotic recombination plays a critical role in sexual reproduction by creating crossovers between homologous chromosomes. These crossovers, along with sister chromatid cohesion, connect homologs to enable proper segregation at Meiosis I. Recombination is initiated by programmed double strand breaks (DSBs) at particular regions of the genome. The meiotic recombination checkpoint uses meiosis-specific modifications to the DSB-induced DNA damage response to provide time to convert these breaks into interhomolog crossovers by delaying entry into Meiosis I until the DSBs have been repaired. The meiosis-specific kinase, Mek1, is a key regulator of meiotic recombination pathway choice, as well as being required for the meiotic recombination checkpoint. The major target of this checkpoint is the meiosis-specific transcription factor, Ndt80, which is essential to express genes necessary for completion of recombination and meiotic progression. The molecular mechanism by which cells monitor meiotic DSB repair to allow entry into Meiosis I with unbroken chromosomes was unknown. Using genetic and biochemical approaches, this work demonstrates that in the presence of DSBs, activated Mek1 binds to Ndt80 and phosphorylates the transcription factor, thus inhibiting DNA binding and preventing Ndt80’s function as a transcriptional activator. Repair of DSBs by recombination reduces Mek1 activity, resulting in removal of the inhibitory Mek1 phosphates. Phosphorylation of Ndt80 by the meiosis-specific kinase, Ime2, then results in fully activated Ndt80. Ndt80 upregulates transcription of its own gene, as well as target genes, resulting in prophase exit and progression through meiosis.

## Author summary

Sexual reproduction requires that cells deliberately introduce large numbers of double strand breaks into their chromosomes. Repair of these breaks creates physical connections between homologs that promote proper segregation during meiosis. It is critical that segregation not proceed until all the breaks have been fixed. How does the cell determine when sufficient double strand break repair has occurred? Our work provides a mechanistic explanation to this question. The meiosis-specific Mek1 kinase is activated by double strand breaks. High numbers of breaks result in high Mek1 activity, resulting in phosphorylation of the meiosis-specific Ndt80 transcription factor. Negative charges conferred by phosphorylation prevent Ndt80 from binding the promoters of its target genes, including genes necessary for completing recombination and meiotic progression, thereby preventing their transcription. As breaks are repaired, Mek1 kinase activity decreases and the inhibitory phosphorylation on Ndt80 is lost, allowing Ndt80 to activate transcription of its target genes. As a result, crossover formation is completed and intact chromosomes proceed properly through the meiotic divisions.

## Introduction

One of the most dangerous things for a cell is the occurrence of DNA double strand breaks (DSBs) in its chromosomes. Failure to repair a DSB may result in a loss of genetic material and lethality. DSBs arise due to exogenous damage such as radiation, or endogenous errors such as stalled replication forks. Repair of DSBs by non-homologous end joining may lead to deletions, translocations or inversions, which can have adverse consequences such as cancer [1]. The most conservative way to repair a DSB is by homologous recombination, using the sister chromatid as the template. Indeed, in mitotically dividing cells, homologous recombination mediated by the evolutionarily conserved recombinase, Rad51, is biased towards using sister chromatids [2, 3].

DSBs trigger an evolutionarily conserved DNA damage checkpoint, which delays or arrests cell cycle progression to provide time for repair [4]. The DNA damage checkpoint is mediated by two kinases, Tel1 (ATM in mammals), which responds to blunt ends, and Mec1 (ATR in mammals) which is activated by single stranded DNA generated by resection of the 5’ ends of the breaks. In yeast, these kinases phosphorylate the adaptor protein, Rad9, which in turn recruits the Forkhead-associated (FHA)-domain containing effector kinase, Rad53, (related to Chk2 in mammals), resulting in Rad53 autophosphorylation and activation. Rad53 phosphorylation of various proteins then prevents cohesin destruction and mitotic exit.

While the purpose of mitosis is to produce genetically identical daughter cells, the specialized cell division of meiosis divides the chromosome number in half to produce gametes for sexual reproduction. After premeiotic chromosome duplication, meiotic chromosomes segregate twice without an intervening round of DNA synthesis: homologous pairs of sister chromatids go to opposite poles at Meiosis I (MI), while sister chromatids separate at Meiosis II (MII). Proper alignment at Metaphase I requires tension that is generated when sister kinetochores from one homolog attach to the spindle pole opposite that of the other homolog. This tension occurs because homologs are physically connected by a combination of crossovers (COs) and sister chromatid cohesion [5]. COs are initiated by programmed DSBs generated by Spo11, a meiosis-specific, evolutionarily conserved topoisomerase-like protein that cuts in preferred regions of the genome called “hotspots” [6]. Unlike mitotic cells, meiotic DSB repair is biased to use the homolog as the repair template [7].

It is key that every pair of homologs contains at least one crossover. Towards this end, many more DSBs are generated during meiotic prophase than the number of necessary COs (e.g.,yeast, 160 DSBs/16 homolog pairs; mouse, 250–300 DSBs for 20 homolog pairs) [6]. The repair of these breaks must be carefully regulated to ensure not only the requisite number of COs, but also that no breaks remain when Anaphase I begins. The meiotic recombination checkpoint delays meiotic prophase while interhomolog recombination is occurring. This checkpoint uses meiosis-specific modifications to the DNA damage checkpoint and is dependent upon protein components of a specialized chromosomal structure called the synaptonemal complex (SC) [8–10].

After chromosome duplication in yeast, cohesin complexes containing the meiosis-specific Rec8 kleisin subunit hold sister chromatids together [11]. Sister chromatids condense along protein cores containing Rec8, as well as the meiosis-specific Hop1 and Red1 proteins, to form axial elements (AEs). Hop1 contains the evolutionarily conserved HORMA domain which mediates homo-oligomerization, as well as interaction with Red1 [12–15]. Chromosome condensation occurs by the formation of chromatin loops, with axis proteins at their bases [16, 17]. Spo11 is indirectly recruited to the axes by phosphorylation of the DSB protein, Mer2 [17–19]. In addition, Mer2 interacts with Spp1, which binds to trimethylated histones flanking hotspot sequences to bring the hotspots to the axis [17, 20, 21]. DSB formation on the loops therefore occurs in the vicinity of Hop1 and Red1 on the axis.

COs created by DSB repair are primarily generated using a functionally diverse set of proteins collectively called the ZMM proteins (Zip1-3, Zip4/Spo22, Msh4, Mer3, Msh5, and Spo16) [22, 23]. Holliday junctions formed by the ZMM pathway exhibit biased resolution to form COs that are distributed throughout the genome [22, 24, 25]. The ZMM pathway is also necessary to form stable associations between homologs, leading to the insertion of the transverse filament protein, Zip1, between the AEs to create the tripartite SC [22, 26, 27]. At the pachytene stage of meiotic prophase, all the homolog pairs are fully synapsed.

Similar to vegetative cells, meiotic DSBs result in the recruitment and activation of the Tel1 and Mec1 checkpoint kinases. These kinases phosphorylate Hop1, which replaces Rad9 as the adaptor [28]. Phosphorylated Hop1 is bound by the FHA domain of the meiosis-specific paralog of Rad53 and Chk2, Mek1 (also known as Mre4), resulting in Mek1 oligomerization and activation by autophosphorylation in *trans* [28–31]. Chromatin-immunoprecipitation experiments using phosphorylation of Histone H3-T11 as a marker for Mek1 activity revealed that this activity is highest at axis sites that correlate with the presence of Hop1 and Red1 and can spread for several kilobasepairs (kb) surrounding a DSB [32].

Mek1 is a key regulator of meiotic DSB repair. It promotes interhomolog bias by inhibiting Rad51 from interacting with its accessory factor, Rad54 in two ways: (1) phosphorylating and stabilizing Hed1, a meiosis-specific protein that binds to Rad51, thereby excluding Rad54 and (2) phosphorylating Rad54 which reduces its affinity for Rad51 [33–36]. These mechanisms prevent Rad51 from competing with the meiosis-specific recombinase, Dmc1, which mediates the bulk of meiotic recombination [37, 38]. Mek1 antagonizes sister chromatid cohesion locally at DSBs to facilitate strand invasion of homologs and regulates whether interhomolog recombination intermediates are repaired as either COs or noncrossovers by enabling phosphorylation of Zip1 by the Cdc7-Dbf4 (DDK) cell cycle kinase [39, 40]. Finally, *MEK1* is required for the meiotic recombination checkpoint delay that prevents cells from entering into the meiotic divisions with unrepaired DSBs [41–44].

Checkpoint delay is part of the normal meiotic program, but this delay can be exacerbated in mutants that initiate, but fail to complete, DSB repair. An extreme case occurs in *dmc1Δ* diploids in the SK1 strain background, where strand invasion does not occur because Dmc1 is absent and Rad51 activity is inhibited by Mek1 [34, 45–47]. The high number of DSBs generates high levels of activated Mek1, resulting in meiotic prophase arrest due to a lack of Cdc28-Clb1 (CDK-Clb1) activity [8, 41, 45, 48, 49]. The checkpoint inhibits CDK-Clb1 by two separate mechanisms: (1) activation and stabilization of the Swe1 kinase which places an inhibitory phosphate on tyrosine 19 of Cdc28 [50] and (2) inactivation of the meiosis-specific transcription factor, Ndt80, thereby preventing *CLB1* transcription [51–53]. During early meiotic prophase, a meiosis-specific E3 ligase targets mitotic regulators such as polo-like kinase (Cdc5) and Clb1 cyclin for degradation [54]. As a result, their production is dependent upon the transcriptional activity of Ndt80.

Ndt80 is a sequence-specific DNA binding protein that recognizes a nine-base pair sequence called the middle sporulation element (MSE) in the promoters of >300 target genes (called “middle” and “late genes”) [51, 55, 56]. *NDT80* transcription occurs in two stages [57]. In the first stage, expression of *NDT80* requires the transcriptional regulator Ime1, which is also responsible for transcribing early genes such as *HOP1*, *MEK1*, *SPO11* and *DMC1* [58]. *NDT80* transcription is delayed relative to the early genes, however, because of the Sum1 repressor, which binds to MSEs in the *NDT80* promoter and the promoters of Ndt80 target genes [59]. Sum1 removal requires phosphorylation by the meiosis-specific Ime2 kinase, in combination with CDK and DDK [60–62]. Since *IME2* is an early gene, it must be transcribed and translated before Sum1 repression can be relieved, hence the delay in Ime1-mediated *NDT80* transcription. The relatively low level of Ndt80 protein generated by Ime1 is inhibited by the meiotic recombination checkpoint until sufficient DSB repair has occurred to lower Mek1 kinase levels below the amount necessary to inactivate Ndt80 [41, 51–53]. The second stage of *NDT80* transcription is marked by phosphorylation of Ndt80 by Ime2 that facilitates Ndt80’s ability to activate transcription [48, 63, 64]. Ndt80 then activates transcription of its own gene to initiate a positive feedback loop, as well as promoting transcription of target genes such as *CDC5*. Expression of *CDC5* triggers to resolution of Holliday junction intermediates into COs and degradation of Red1 to dissemble the SC [41, 54, 65]. Removal of Red1 leads to inactivation of the remaining Mek1, allowing residual DSBs to be repaired prior to *CLB1*-promoted entry into Meiosis I [41].

Exit from pachynema and entry into Meiosis I has been proposed to be controlled by a switch between two stable states [54]. In the first state, CDK-Clb1 levels are low due to the meiotic recombination checkpoint, thereby preventing meiotic progression. In the second state, CDK-Clb1 levels are high because DSBs have been repaired, leading to a decrease in the checkpoint signal and activation of Ndt80, thereby allowing *CLB1* transcription and progression into the meiotic divisions. What was unknown was how this switch is controlled. This work shows that Mek1, after being activated by DSBs, directly binds and phosphorylates Ndt80, thereby inhibiting Ndt80 from activating transcription. As DSBs are repaired, Mek1 activity decreases, and inhibitory Mek1 phosphosites are removed. Ime2 phosphorylation then promotes Ndt80 activity, resulting in expression of genes necessary for completing recombination and exiting prophase. Mek1 phosphorylation of Ndt80 therefore provides an elegant way for cells to know when it is safe to enter the first meiotic division.

## Results

### Mek1 interacts with a conserved sequence within a domain of Ndt80 that is required for meiotic recombination checkpoint arrest

**Fig 1:**
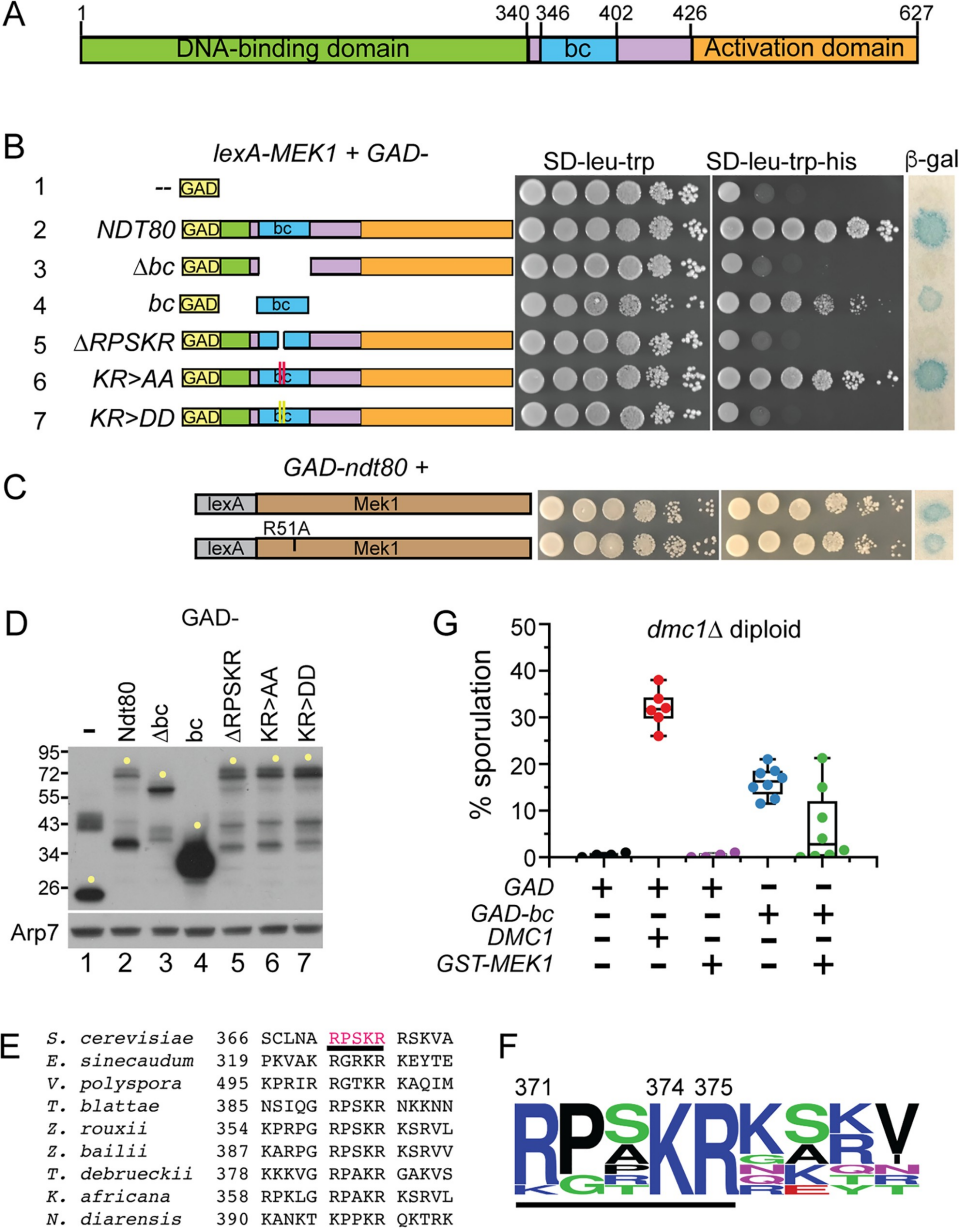
Mek1 interaction with Ndt80 in the two-hybrid system involves a conserved five amino acid sequence in the *bc* domain. (A) Domains of Ndt80. Green represents the DNA binding domain [67, 68], blue indicates the 57 amino acid “*bc*” sequence that is required for meiotic recombination checkpoint arrest [69] and orange indicates the transcriptional activation domain [64]. (B) Two-hybrid interactions between *lexA-MEK1* and various *GAD*-*NDT80*^*287-627*^ fusions (for simplicity, this fusion is called *GAD-NDT80*). The *lexA-MEK1* allele on pTS3 was co-transformed with plasmids containing *GAD* (pACTII), *GAD-NDT80* (pXC13), *GAD- NDT80-Δbc* (pXC14), *GAD-bc* (pXC18), *GAD-NDT80-ΔRPSKR* (pNH318), *GAD-NDT80-KR>AA* (pXC13-AA) or *GAD-NDT80*^*-*^*KR>DD* (pXC13-DD) into L40 and assayed for *HIS3* and *lacZ* expression. Alanine and aspartic acid mutations are indicated by red and yellow vertical lines, respectively. (C) *GAD-NDT80* was co-transformed with either *lexA-MEK1* or *lexA-mek1-R51A* (pTS3-R51A) and two-hybrid interactions were measured as in Panel B. (D) Immunoblot detecting the GAD fusion proteins assayed in Panel B. Extracts from the same cultures used for the spotting assays were probed with antibodies to either GAD or Arp7 (as a loading control). Yellow dots indicate full-length proteins. (E) Alignment of the RPSKR region from *S*. *cerevisiae* Ndt80 with Ndt80 proteins from related fungi. Numbers indicate the amino acid positions. The underlined red sequence was deleted for two-hybrid and functional experiments. (F) Consensus motif based on the alignment in E. The motif was generated using http://weblogo.threeplusone.com/ [71]. Basic amino acids are blue, polar are green, neutral are purple and hydrophobic are black. The five amino acids deleted in *GAD-ndt80-ΔRSPKR* are underlined. Numbers indicate the amino acid position in Ndt80. (G) Sporulation in a *dmc1Δ* diploid overexpressing *GAD-bc*. NH2444 was transformed with 2μ *GAD* (pACTII/pRS316), 2μ *GAD*, *CEN ARS DMC1* (pACTII/pRS316-DMC1), 2μ *GAD*, 2μ *GST-MEK1* (pACTII/pLW1), 2μ *GAD-bc* (pLB1/pRS316), or 2μ *GAD-bc*, 2μ *GST-MEK1* (pLB1/pLW1). Each dot represents an independent transformant. Whisker plots indicate the medians and standard deviations.

A two-hybrid screen using *lexA*-*MEK1* revealed an interaction with a fragment of *NDT80* (amino acids 287–627) fused to the Gal4 activation domain (GAD). This fusion is hereafter referred to as *GAD-NDT80*. The strain contained *HIS3* and *lacZ* reporter genes under the control of promoters containing *lexA* operator sites [66]. Two-hybrid interactions were therefore manifested either by growth on medium lacking histidine or production of ß -galactosidase. The *GAD-NDT80* fragment begins near the end of the Ndt80 DNA binding domain (DBD) and goes to the end of the protein. In addition to the activation domain in the C terminus, this fragment includes a 57 amino acid sequence in the middle of Ndt80 that is required for meiotic recombination checkpoint arrest (Fig 1A and 1B, row 2)[64, 67–69]. The *NDT80-bc* allele, which encodes an Ndt80 protein deleted for this 57 amino acid sequence, no longer responds to the checkpoint triggered by unrepaired breaks in both *zip1Δ* and *dmc1Δ* mutants (bc stands for “bypass checkpoint”)[69]. We have therefore named this 57 amino acid sequence the “bc” domain. Disruption of the Mek1 FHA domain using the R51A mutation had no effect on the Ndt80 interaction, indicating that the FHA domain does not mediate binding (Fig 1C)[47, 70]. In contrast, deletion of the *bc* domain from *GAD-NDT80* eliminated interaction with *lexA-MEK1*, even though the GAD-Ndt80-Δbc protein was more abundant than GAD-Ndt80, ruling out protein instability as the reason for the loss of the two-hybrid signal (Fig 1B, row 3 and 1D, lanes 2 and 3). In addition to being necessary for *lexA-MEK1* interaction, the *bc* domain was also sufficient, as the *GAD-bc* fusion produced a positive two-hybrid signal in combination with *lexA-MEK1* (Fig 1B, row 4).

A 60 amino acid sequence containing the *bc* domain was used to probe Ndt80 proteins from other fungi for homology. A small region containing amino acids 371–375, RPSKR, is conserved in several yeast species (Fig 1E). A consensus motif generated from these alignments showed that lysine (K) 374 and arginine (R) 375 from *S*. *cerevisiae* Ndt80 are completely conserved (Fig 1F). Deletion of the sequence encoding RPSKR from *GAD-NDT80* abolished the two-hybrid interaction with *lexA-MEK1*, as did substituting the KR sequence with aspartic acids (DD) (Fig 1B, rows 5 and 7). The *KR* to alanine (*AA*) mutant still interacted with *lexA-MEK1*, although not quite as well as *GAD-NDT80* (Fig 1B, compare rows 6 and 2). The RPSKR sequence is therefore required for interaction between lexA-Mek1 and Ndt80.

### Over-expression of the *NDT80 bc* domain partially bypasses the meiotic recombination checkpoint in *dmc1Δ* diploids

The Ndt80-Mek1 interaction has thus far not been confirmed by co-immunoprecipitation experiments from meiotic extracts due to technical problems obtaining soluble Ndt80. A functional genetic approach was therefore used to test the importance of this interaction *in vivo*. Overexpression of *NDT80* can partially bypass the meiotic recombination checkpoint arrest triggered by the unrepaired DSBs that accumulate when the *DMC1* recombinase is absent [53, 69, 72]. One explanation for this result is that during meiosis in wild-type (WT) cells there is sufficient Mek1 to bind and inactivate all of the Ime1-dependent Ndt80 protein. However, when Ndt80 is in excess of Mek1, some Ndt80 escapes phosphorylation, resulting in transcription of the *NDT80* gene to start the positive feedback loop leading to meiotic progression. If this model is correct, and if the *bc* domain recruits Mek1 to Ndt80 in *dmc1Δ*-arrested cells, then over-expressing the *bc* domain by itself could titrate Mek1 away from endogenous Ndt80, resulting in activation of the transcription factor and sporulation. To limit expression of the *GAD-bc* fusion to meiotic cells, the hybrid gene was placed under the control of the *MEK1* promoter. A *dmc1Δ* diploid transformed with a *DMC1 CEN ARS* plasmid only partially complemented the sporulation defect, perhaps due to plasmid loss during growth on the Spo plate (Fig 1G). The *GAD-bc* transformants partially bypassed the *dmc1Δ* checkpoint arrest, exhibiting increased sporulation after three days on Spo medium compared to *GAD* alone (Fig 1G). Further support for the titration model is that this partial checkpoint bypass was decreased when counteracted by overexpression of *GST-MEK1* from a high copy number plasmid (Fig 1G).

#### Ndt80 interaction with Mek1 is required for meiotic recombination checkpoint arrest

**Fig 2:**
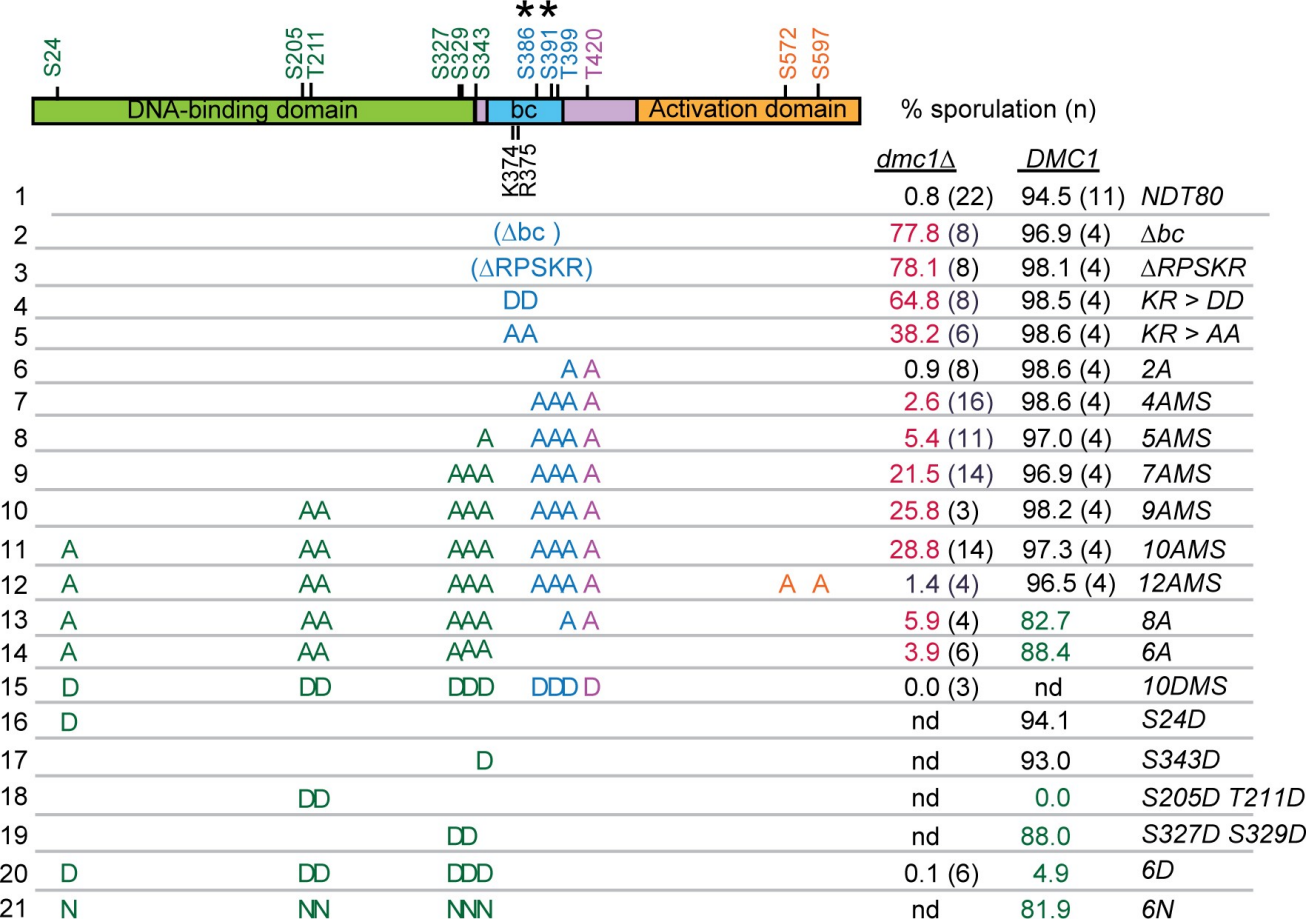
Sporulation analysis of various *ndt80* mutants in *dmc1Δ* and *DMC1* diploids. Potential Mek1 phosphorylation sites are indicated in green, purple and gold, corresponding to the protein domain and have the sequence RXXT/S. Asterisks indicate non-consensus sites detected as phosphorylated in global phosphoproteomic analyses of *dmc1Δ*- arrested cells [73]. Sporulation was assayed after three days at 30ºC on solid sporulation medium. Each row represents a diploid homozygous for a different allele of *NDT80* with mutated residues indicated by either A (alanine), D (aspartic acid) or N (asparagine). “nd” indicates no data. (*NDT80*, pHL8; *NDT80-Δbc*, pHL8-*Δ*bc; *ndt80-ΔRPSKR*, pNH317; *ndt80-KR>AA*, pHL8-KR>AA; *ndt80-KR>DD*, pHL8-KR>DD; *ndt80-2A*, pHL8-2A; *ndt80-4AMS*, pHL8-4AMS; *ndt80-5AMS*; pHL8-5AMS; *ndt80-7AMS*, pHL8-7AMS; *ndt80-9AMS*, pHL8-9AMS; *ndt80-10AMS*, pHL8-10AMS; *ndt80-8A*, pNH405; *ndt80-6A*, pNH400; *ndt80-10DMS*, pHL8-10DMS; *ndt80-S24D*, pHL8-S24D; *ndt80-S343D*, pHL8-S343D; *ndt80-S205D T211D*, pHL8-S205D T211D; *ndt80-S327D S329D*, pHL8-S327D S329D; *ndt80-6D*, pNH401; *ndt80-6N*, pHL8-6N. Sporulation was scored in either a *dmc1Δ* (NH2402) or *DMC1* diploid (NH2081). Values in magenta are significantly higher than *dmc1Δ NDT80* with *p* values <9 X10^-10^, while values in green are significantly lower than *DMC1 NDT80* with *p* values < 0.014 (*ndt80-6A*) or 9.58 X 10^−8^. *p* values were determined using a one-sided Fisher’s Exact Test. Strains, standard deviations, number of biological replicates and *p* values are listed in Data S1.

The ability of *GAD-NDT80* to interact with *lexA-MEK1* could be related to how Ndt80 is inactivated by Mek1 in *dmc1Δ*-arrested cells. Deletion of either the *bc* or *RPSKR* sequence from *NDT80*, as well as *ndt80*-*KR>DD*, rescued the sporulation defect of *dmc1Δ* (Fig 2, compare row 1 to 2–4) and produced inviable spores (spore viability: *Δbc*, 2.6% ± 3.7, 118 asci; *ΔRPSKR*, 7.5% ± 5.0; 80 asci; *KR>DD*, 3.9% ± 2.7, 160 asci). Spore inviability was expected as activation of *CDC5* transcription by Ndt80 results in Red1 degradation, inactivation of Mek1 and repair of DSB using sister chromatids, rather than homologs [41]. Bypass of the *dmc1Δ* checkpoint arrest by *ndt80-KR>AA* was reduced relative to the *DD* mutant, consistent with its increased ability to interact with *lexA-MEK1*, but still produced mostly inviable spores (6.7 ± 6.2, 120 asci)(Fig 2, rows 4 and 5).

**Fig 3:**
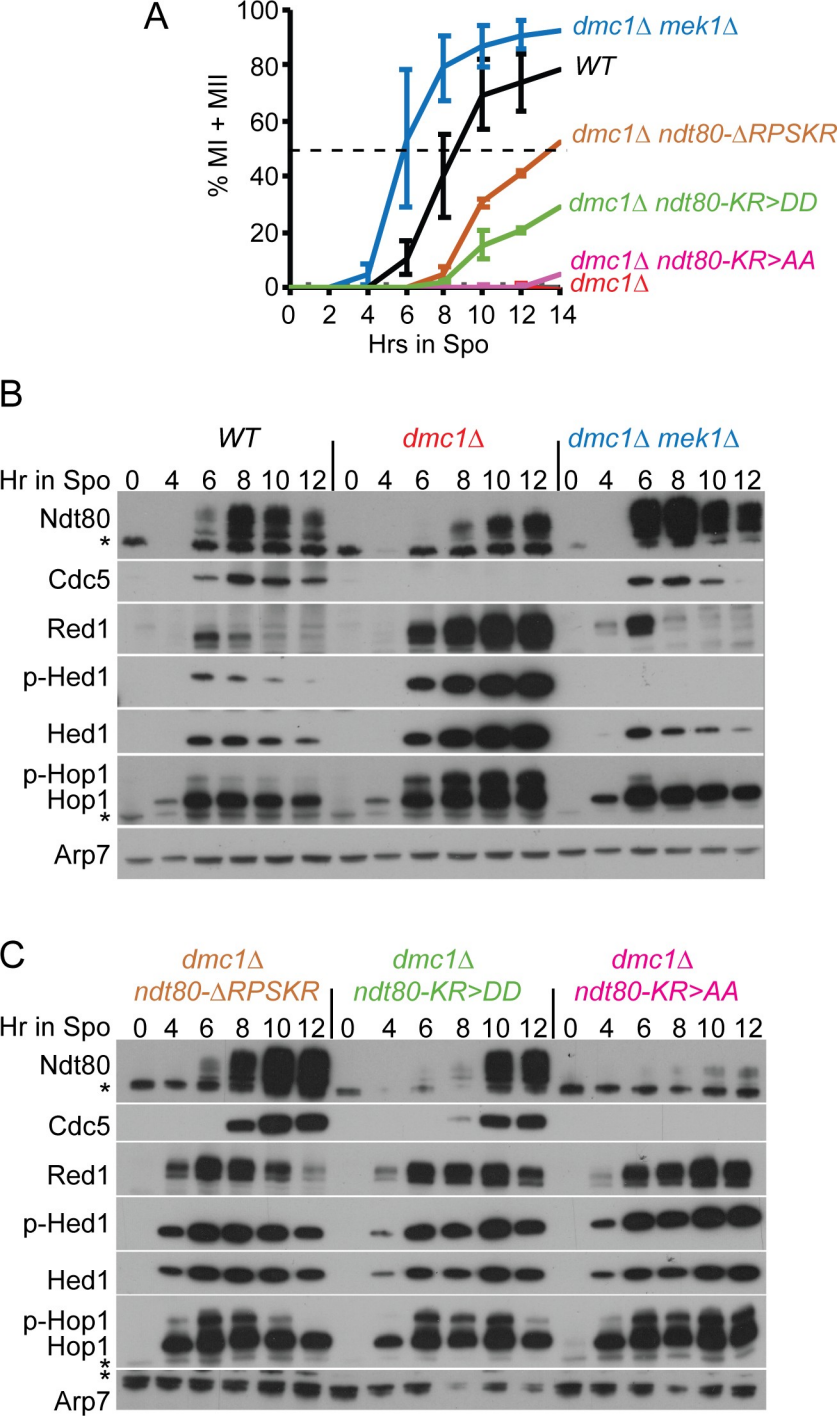
Mek1 interaction-defective *ndt80* mutants bypass the *dmc1Δ* meiotic recombination checkpoint arrest. WT (NH2081::pHL8^2^), *dmc1Δ*, (NH2402::pHL8^2^), *dmc1Δ mek1Δ* (NH749), *dmc1Δ ndt80-ΔRPKSR* (NH2402::pNH317^2^), *dmc1Δ ndt80-KR>AA* (NH2402::pHL8-KR>AA^2^) and *dmc1Δ ndt80-KR>DD* (NH2402::pHL8-KR>DD^2^) were transferred to Spo medium at 30°C and cells analyzed at the indicated timepoints. (A) Meiotic progression. The percent of cells completing either MI or MII was determined using fluorescence microscopy of DAPI-stained nuclei. Two hundred cells were counted per timepoint. The average values of two experiments were plotted with error bars indicating the range. (B) Immunoblot analysis of extracts from WT, *dmc1Δ* and *dmc1Δ mek1Δ* diploids taken at the indicated timepoints from one of the timecourses shown in A. (C) Immunoblot analysis of extracts from *dmc1Δ ndt80-ΔRPSKR*, *dmc1Δ ndt80-KR>DD* and *dmc1Δ ndt80-KR>AA* diploids taken at the indicated timepoints from one of the timecourses shown in A. Phospho-Hop1 (p-Hop1) is an indirect indicator of DSBs, while phospho-Hed1 T40 (p-Hed1) is a marker for Mek1 kinase activity. The asterisks indicate non-specific bands. Arp7 was used as a loading control.

If the RPSKR sequence in Ndt80 is important for interaction with Mek1 in meiotic cells, then mutation of this site should be specifically defective in the checkpoint function of Mek1, while allowing Mek1 to phosphorylate its other targets. This is in contrast to *mek1Δ*, where all Mek1 phosphorylation is eliminated, including checkpoint targets such as Ndt80, as well as recombination regulators like Hed1 and Rad54. This hypothesis was tested by meiotic time course analysis of *dmc1Δ* diploids containing either *mek1Δ* or *ndt80-ΔRPSKR*. While both mutants efficiently suppressed the sporulation defect of *dmc1Δ* when given enough time (Fig 2, row 3)[30], the kinetics of meiotic progression were dramatically different. The *dmc1Δ mek1Δ* diploid progressed faster through meiosis than *DMC1 MEK1* (consistent with the literature), while meiotic progression for *dmc1Δ ndt80-ΔRPSKR* was slower (Fig 3A) [74].

The rate of meiotic progression correlated with the appearance of active Ndt80, which can be determined based on the increased abundance of Ndt80 and its increased level of phosphorylation due to Ime2, as well as the production of Cdc5. In WT cells, peak levels of these two proteins were observed after eight hours in Spo medium, when approximately ~40% of the cells had completed either MI or MII (Fig 3A and 3B). In *dmc1Δ*, Hop1 and Hed1 T40 phosphorylation, which are indirect indicators of DSBs and Mek1 kinase activity, respectively, persisted up to 12 hours in Spo medium with no meiotic progression (Fig 3A and 3B). Ndt80 was present but exhibited a reduced level of phosphorylation. No Cdc5 was detected, confirming that Ndt80 was inactive. In contrast, active Ndt80 was detected by six hours in the *dmc1Δ mek1Δ* strain by which time ~55% of the cells has entered the meiotic divisions (Fig 3A and 3B). Hop1 phosphorylation was nearly gone by six hours, indicating DSB repair had occurred (Fig 3B). The absence of Mek1 kinase activity was confirmed by lack of Hed1 phosphorylation, which resulted in degradation of Hed1 (Fig 3B) [34].

The *ndt80-ΔRPSKR* diploid exhibited phenotypes predicted for a mutant that is defective in meiotic recombination checkpoint arrest at a step downstream of Mek1 activation. Highly phosphorylated Ndt80 and Cdc5 were present by 8 hours, even though Hop1 phosphorylation and Mek1 activity persisted (Fig 3B). This is the essence of a checkpoint bypass—while the signal to trigger the checkpoint was still present, the inability of Mek1 to bind to Ndt80 allowed the transcription factor to become active, as evidenced by the production of Ndt80 and Cdc5.

The *ndt80-KR>AA* and *DD* mutants provide further evidence for the correlation between Mek1 interaction and meiotic recombination checkpoint activity. *GAD-NDT80-KR>DD* exhibited no *lexA-MEK1* interaction, while *GAD-NDT80-KR>AA* exhibited binding to *lexA-MEK1*, though not as strongly as *GAD-NDT80* (Fig 1B). These differences were mirrored in the time course results. *ndt80-KR>DD* progressed through meiosis more quickly than *NDT80-KR-AA*, but more slowly than *NDT80-ΔRPSKR* (Fig 3A). This result is consistent with RPSKR deletion reducing Ndt80’s affinity for Mek1 more than the KR to DD substitutions. In the *NDT80-KR>DD* diploid, Ndt80 phosphorylation and Cdc5 did not peak until 10 hours. Similar to *NDT80-ΔRPSKR*, Hop1 and Hed1 phosphorylation levels were high at this time, indicating a bypass of the checkpoint. In contrast, in the *NDT80-KR>AA* mutant, highly phosphorylated Ndt80 and Cdc5 were not detected even after 12 hours in Spo medium (Fig 3B). We conclude that Mek1 interaction with the Ndt80 RPSKR sequence is necessary to efficiently inactivate Ndt80.

### Disrupting the Ndt80-Mek1 interaction reveals a role for Mek1 in protecting Red1 from degradation

Previous work has shown that the presence of Cdc5 is sufficient to trigger SC disassembly and Red1 degradation [41, 54, 65, 75]. These experiments showed that induction of *CDC5* in the *ndt80Δ* background (where Mek1 levels are low) resulted in the disappearance of Red1 and elimination of the SC [41, 65]. Interfering with the Mek1-Ndt80 interaction in the *dmc1Δ* background allowed activation of Ndt80 (indicated by increased phosphorylation) and production of Cdc5 in the presence of high levels of Mek1 activity (Fig 3C, *dmc1Δ ndt80-ΔRPSKR* and *dmc1Δ ndt80-KR>DD*). In these cases, Red1 persisted for at least two hours after Cdc5 was first detected. In contrast, Red1 was eliminated within two hours after the appearance of Cdc5 in the *dmc1Δ mek1Δ* diploid (Fig 3C). These results suggest that Cdc5 is not as efficient in targeting the degradation of Red1 in the presence of high levels of Mek1 activity (Fig 3B). Red1 disappears more rapidly in the *dmc1Δ ndt80-ΔRPSKR* mutant than *dmc1Δ ndt80-KR>DD* and persisted for the length of the time course in the *dmc1Δ ndt80-KR>AA* strain (Fig 3C). These differences reflect the larger defect in Mek1 interaction resulting from the deletion of the RPSKR sequence compared to the aspartic acid substitution mutations. Ndt80 was activated more quickly in *ndt80-ΔRPSKR* (increased phosphorylation at 8 hours) (Fig 3C) so Cdc5 was produced earlier as well. While Mek1 kinase activity delayed Red1 degradation in the presence of Cdc5, it did not prevent it completely. As a result, Mek1 was gradually decreased due to loss of Red1, allowing DSB repair by Rad51 (indicated by loss of Hop1 phosphorylation), leading to a further reduction in Mek1 activity and more efficient Cdc5-dependent degradation of Red1 (Fig 3C). These results have therefore uncovered yet another mechanism to ensure that cells do not enter MI prematurely, i.e., the prevention of Red1 degradation and therefore, SC disassembly, when Mek1 levels are high. The mechanism by which Mek1 inhibits Red1 degradation remains to be determined.

#### Ndt80 activity is downregulated by the presence of negative charges at putative Mek1 consensus phosphorylation sites

The discovery that Mek1 and Ndt80 physically interact suggests that Ndt80 activity is inhibited by Mek1 phosphorylation. *In vivo* Mek1 substrates (Mek1-T327, Rad54-T142, Hed1-T40 and histone H3-T11) as well as analysis of phosphoproteomic datasets from *dmc1Δ*-arrested cells, indicate that the preferred consensus site for Mek1 *in vivo* is RXXT [30, 32, 34, 35, 73]. However, *in vitro* Mek1 preferentially phosphorylates both RXXT and RXXS [76]. There are ten RXXS/T sites in Ndt80, five in the DNA binding domain (DBD), three in the middle region and two in the activation domain (Fig 2). In addition, two phosphorylated amino acids within the *bc* domain were detected by mass spectrometry (MS) analysis of chromatin-associated proteins from *dmc1Δ*-arrested cells (indicated by asterisks in Fig 2) [73]. Whether the non-consensus MS phosphosites are due to Mek1 or another kinase has not yet been determined. The hypothesis that phosphorylation of Mek1 consensus sites and/or the MS-identified serines inhibits Ndt80 activity was tested using non-phosphorylatable alanine substitutions and assaying for bypass of *dmc1Δ*-mediated meiotic recombination checkpoint arrest.

Alanine substitutions of the two Mek1 consensus sites within the middle region of Ndt80, T399 and T420, (*NDT80-2A*) behaved like *NDT80* in the *dmc1Δ* background and did not sporulate (Fig 2, row 6). Combining mutations in the *in vivo* phosphorylated non-consensus sites (S386 and S391) with T399A and T420A (*NDT80-4AMS*) resulted in a small, but statistically significant, increase in sporulation compared to the *dmc1Δ NDT80* diploid (Fig 2, row 7). An increase in Ndt80 activity (indicated indirectly by the ability of cells to sporulate) was observed with increasing numbers of alanine mutations in Mek1 consensus sites located within the DBD (Fig 2, rows 8–11). The *NDT80-10AMS* allele exhibited the strongest checkpoint bypass with 28.8% sporulation (Fig 2, row 11). One explanation for the partial bypass observed for *NDT80-10AMS* is that the mutations make the transcription factor less effective. In fact, alanine substitutions at all 10 Mek1 consensus sites plus the two MS (*NDT80-12AMS*) did not increase the *dmc1Δ* bypass, but instead had the opposite effect, resulting in very low sporulation (Fig 2, row 12). If mutational load is a problem, it is only manifested under *dmc1Δ* checkpoint-arrested conditions, however, as the *NDT80-10AMS* and *-12AMS* diploids sporulated like WT in the *DMC1* background.

The *NDT80-8A* mutant contains alanine substitutions in the same eight Mek1 consensus sites that were changed in *NDT80-10AMS*, but allows phosphorylation of the MS sites, S386 and S391. *NDT80-8A* cells arrested more efficiently than *NDT80-10AMS*, indicating that phosphorylation of non-consensus Mek1 sites also contributes to Ndt80 inactivation (Fig 2, row 13).

One feature of phosphorylation is that negative charges are added onto localized regions of a protein. The bypass observed for the *ndt80* alanine mutants could be due to a lack of these negative charges, or it could be that serine and/or threonine are required for some other reason. To distinguish between these possibilities, putative Mek1 phosphosites were substituted with aspartic acid (D). The negative charge of aspartic acid can sometimes mimic phosphorylation, which in this situation would create a constitutively inactive version of *NDT80*. *NDT80-10DMS* completely failed to sporulate in the *dmc1Δ* diploid, providing genetic evidence that the 10AMS phenotype is due to a lack of phosphorylation (Fig 2, row 15).

Substituting aspartic acid for the serines and threonines in the Mek1 consensus sites located within the Ndt80 DBD (*ndt80-6D*) was sufficient to constitutively inactivate Ndt80, reducing sporulation in a *DMC1* diploid from 91.6% to 4.9% (Fig 2, row 20). (It should be noted that S343 is on the border between the DNA binding domain and the middle region and therefore may not be part of the DBD *per se*). Individual aspartic acid substitutions at S24 and S343 did not reduce sporulation in the *DMC1* background, while the *ndt80-S327D S329D* mutant exhibited only a modest reduction (Fig 2, rows 16, 17 and 19). In contrast, negative charges at S205 and T211 (*ndt80-S205D T211D*) constitutively inactivated Ndt80 in a *DMC1* diploid (Fig2, row 18).

One caveat in interpreting the phenotype of *NDT80* alleles containing aspartic acid substitutions is that the aspartic acid side chain is longer than serine/threonine side chains, and this difference in length could be disrupting Ndt80 function, as opposed to the presence of the negative charges. The side chain of asparagine (N) is similar to aspartic acid, except that an uncharged amide group replaces a negatively charged carboxylate group. If charge, and not length, is responsible for inactivating *ndt80-6D*, the *NDT80-6N* mutant should be active. This was indeed the case, as the *NDT80-6N* diploid exhibited 81.9% sporulation (Fig 2, row 21). These results support a model by which negative charges conferred by phosphorylation of the Ndt80 DBD inactivate the transcription factor.

### Negative charges on the Ndt80 DBD prevent meiotic progression and expression of Ndt80 target genes

**Fig 4:**
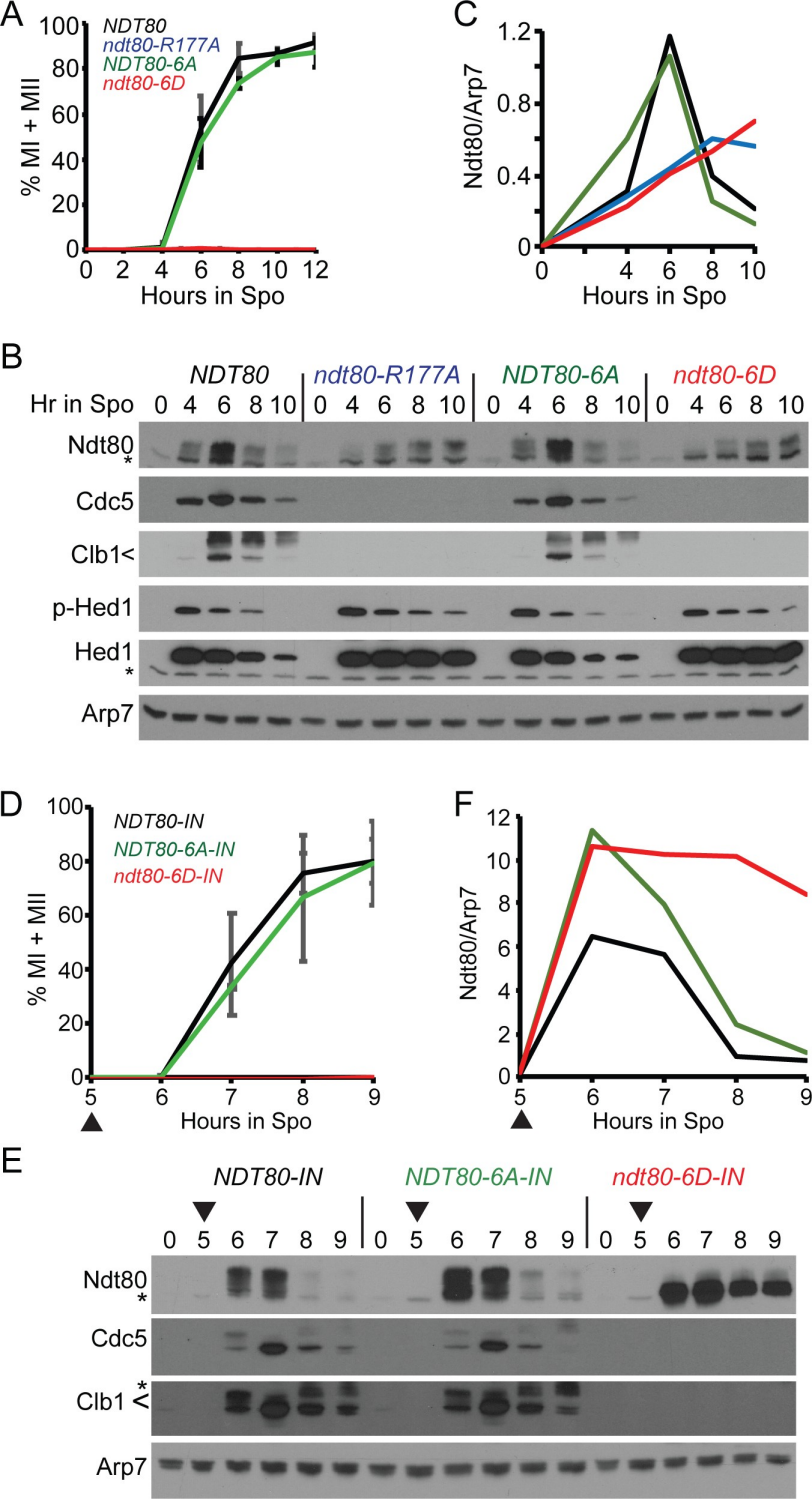
Negatively charged mutations in the Ndt80 DNA binding domain constitutively inactivate Ndt80. (A-C) Endogenous *NDT80*. (A) Meiotic progression. Cells expressing *NDT80* (NH2426::pEP105^2^::pHL8^2^), *ndt80-R177A* (NH2426::pEP105^2^::pHL8-R177A^2^), *ndt80-6A* (NH2426::pEP105^2^::pNH400^2^), and *ndt80-6D* (NH2426::pEP105^2^::pNH401^2^) were transferred to Spo medium to induce sporulation. Meiotic progression was determined as described in Fig 3 using three independent timecourses with error bars indicating the standard deviations. (B) Immunoblot analysis of protein extracts from one of the timecourses used in (A). (C) Quantification of Ndt80 signal in (B). Ndt80 was normalized to Arp7 from the same lane on the same gel. The signal from non-specific bands was eliminated by subtracting the Ndt80/Arp7 from 0 hours from each timepoint of the appropriate strain. (D-F) Inducible *NDT80*. (D) Meiotic progression. Cells expressing *NDT80-IN* (NH2426::pEP105^2^::pBG4^2^), *ndt80-6A-IN* (NH2426::pEP105^2^::pXC11^2^), and *ndt80-6D-IN* (NH2426::pEP105^2^::pXC12^2^) were transferred to Spo medium and incubated for five hours. *NDT80* expression was induced by addition of estradiol (ED) to a final concentration of 1 μM (indicated by arrowheads). Data represent the average values of two experiments with error bars indicating the ranges. (E) Immunoblot analysis of proteins extracts obtained from one of the timecourses used in (D). (F) Ndt80 quantification from (E).

Meiotic time courses were performed with *NDT80*, *NDT80-6A*, *ndt80-6D* and *ndt80-R177A* diploids. The *ndt80-R177A* diploid was used as a negative control as the Ndt80-R177A protein is defective in binding to MSEs and therefore is unable to activate transcription either of itself or other *NDT80* targets [61, 67, 68, 77]. The *ndt80-6D* diploid was phenotypically identical to *ndt80-R177A*, indicating that it also is defective in activating transcription. Both mutants arrested in meiotic prophase, while *NDT80* and *NDT80-6A* exhibited similar kinetics for meiotic progression (Fig 4A). The *R177A* and *6D* diploids entered the meiotic program efficiently, as evidenced by similar levels of phosphorylated Hed1 protein compared to *NDT80* and *NDT80-6A* at the 4-hour time point (Fig 4B)[34]. Whereas the Ndt80 and Ndt80-6A protein levels peaked at six hours and then decreased until they were nearly gone by 10 hours, the R177A and 6D proteins exhibited reduced levels that slowly accumulated throughout the length of the time course (Fig 4B and 4C). This result is consistent with the occurrence of Ime1-driven transcription of the *ndt80-R177A* and *ndt80-6D* genes, followed by a failure of the mutant proteins to activate transcription of their own genes. In addition, the *R177A* and *6D* mutants failed to express *CLB1* and *CDC5*, although both proteins were observed for *NDT80* and *NDT80-6A* (Fig 4B).

An alternative explanation for the *ndt80-6D* phenotypes is that Ndt80-6D is transcriptionally active, but the aspartic acid substitutions destabilize the protein so that there is insufficient Ndt80-6D protein to promote transcription of *CDC5*, *CLB1*, etc. This hypothesis was tested using *ndt80-6D* under control of the *GAL1* promoter in a strain containing a *GAL4*-estrogen receptor fusion (*GAL4-ER*). The resulting allele (indicated as *ndt80-6D-IN*) can be induced ectopically by addition of estradiol to the Spo medium [41, 48, 78]. If the aspartic acid residues destabilize Ndt80, then the induced Ndt80-6D levels should be lower compared to Ndt80 and Ndt80-6A. In contrast, if the reduced level of the endogenous Ndt80-6D protein is due to a failure in Ndt80-activated transcription of the *ndt80-6D* gene, Ndt80-6D levels should be equivalent to Ndt80 and Ndt80-6A, since transcription is now under the control of a heterologous promoter.

Induction of the *NDT80-IN* alleles after five hours in Spo medium resulted in meiotic progression of the *NDT80* and *NDT80-6A* diploids, while *ndt80-6D* remained arrested in prophase (Fig 4D). Both the Ndt80-6A and Ndt80-6D proteins were present in greater abundance than Ndt80 throughout the timecourse. Importantly, the Ndt80-6A and Ndt80-6D proteins exhibited similar kinetics of induction and peaked at the same level. However, while the Ndt80-6A protein was nearly gone by 9 hours, the Ndt80-6D protein persisted and exhibited reduced phosphorylation (Fig 4E and 4F). Cdc5 and Clb1 were generated in the WT and *NDT80-6A* strains but not in *ndt80-6D*, confirming that constitutive negative charges at Mek1 consensus sites in the DBD impede the ability of Ndt80 to activate transcription (Fig 4E).

### Ndt80 is phosphorylated in its inactive state independently of *IME2*

Phosphorylation of Ndt80 by Ime2 results in multiple mobility shifts that enhance Ndt80 transcriptional activity [48, 63, 64]. One report found that inactive Ndt80 derived from checkpoint arrested cells was not phosphorylated [53], and suggested that Ndt80 phosphorylation is solely used for activation of the transcription factor. A different group detected a phosphorylation-dependent mobility shift in a *dmc1Δ* diploid which was not as slow as the Ndt80 mobility shifts observed from WT cells [63], consistent with our hypothesis that Mek1 phosphorylation of Ndt80 is inhibitory. One difficulty with interpreting these experiments is that the checkpoint prevents Ndt80 from activating transcription of itself, and therefore Ndt80 protein levels are low, making the protein more difficult to detect [51–53]. The estradiol-inducible *NDT80* system was therefore used to determine whether inactive Ndt80 is phosphorylated.

**Fig 5:**
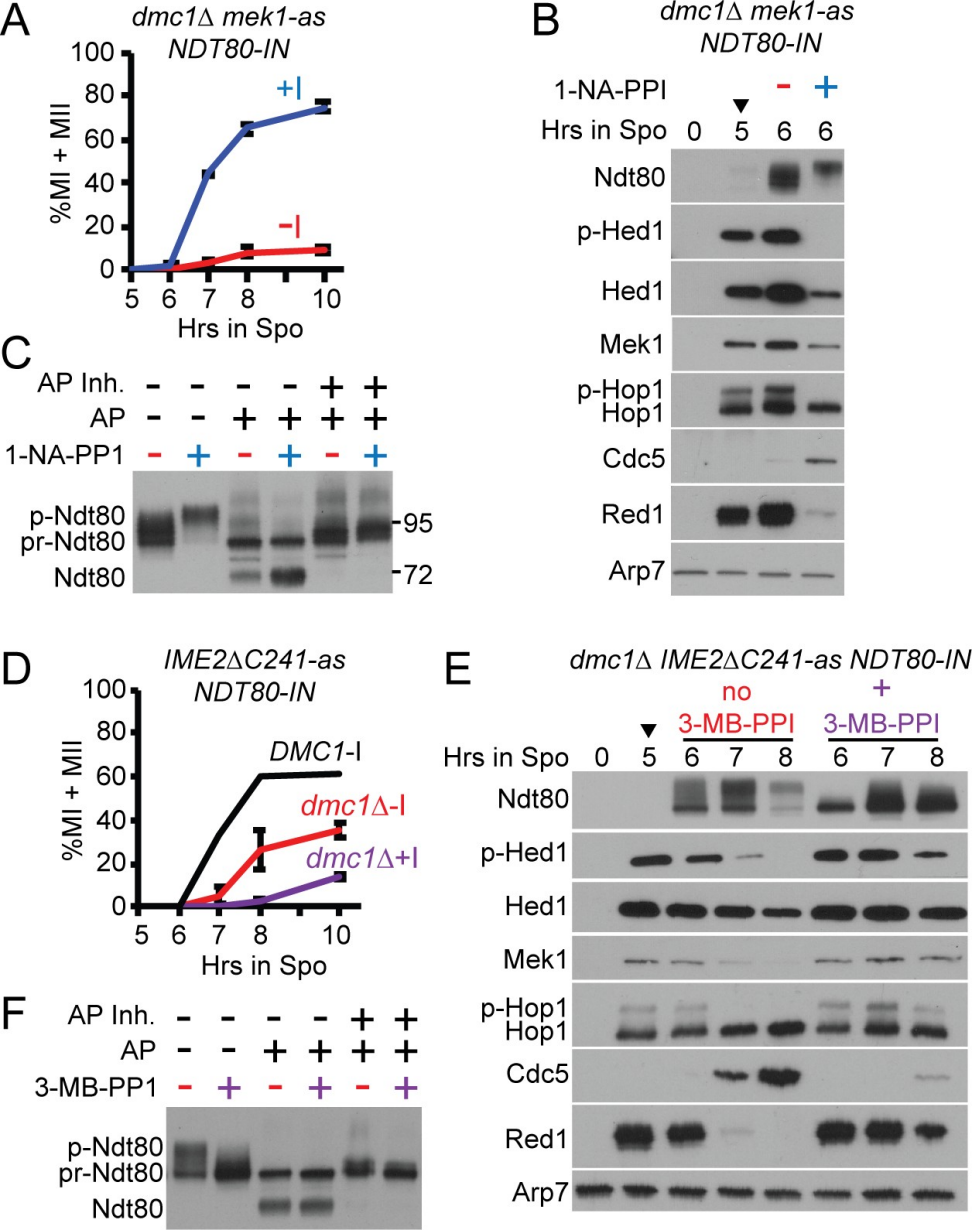
Phosphatase treatment of Ndt80 with and without Mek1-as or Ime2ΔC241-as kinase activity. For all of the experiments in this figure, diploids were transferred to Spo medium for five hours, 1 μM ED was added (indicated by arrowheads) and the culture split in two. Either 1 μM 1-NA-PP1 (*mek1-as*) or 50 μM 3-MB-PP1 (*IME2ΔC241-as*) was added to one of the cultures and samples assayed at various timepoints. (A) Meiotic progression in *dmc1Δ mek1-as NDT80-IN* (NH2437::pEP105^2^::pBG4^2^) without (-I) and with (+I) 1-NA-PP1. Values represent the average of two independent experiments with error bars indicating the range. (B) Extracts generated from cells at the 0, 5 and 6 hr (- and + I) timepoints from one of the timecourses shown in (A) probed with antibodies recognizing the indicated proteins. (C) Phosphatase treatment of the 6 hr (- and +I) extracts. AP = alkaline phosphatase. p-Ndt80 indicates phosphorylated Ndt80; pr-Ndt80 = phosphatase resistant form of Ndt80; Ndt80 = unphosphorylated Ndt80. AP was preincubated with phosphatase inhibitors (AP Inh.) for 30 minutes prior to addition to extracts. Numbers indicate where the prestained molecular weight markers (in kD) ran on the gel. (D) Meiotic progression in *DMC1 IME2ΔC241-as NDT80-IN* and *dmc1Δ IME2ΔC241-as NDT80-IN* (yJL92 and NH2451, respectively) without (-I) and with (+I) 3-MB-PP1 as in Panel A. (E) Extracts from the indicated timepoints from one of the *dmc1Δ IME2ΔC241-as NDT80-IN* timecourses shown in D probed with antibodies recognizing the indicated proteins. (F) Phosphatase treatment of the 7 hour extracts shown in (E).

A *dmc1Δ mek1-as NDT80-IN* diploid was incubated in Spo medium for 5 hours to arrest cells with unrepaired DSBs. The *mek1-as* allele encodes an analog-sensitive (*as*) kinase with an enlarged ATP binding pocket that allows for inhibition of the kinase by addition of the 1-NA-PP1 inhibitor to the Spo medium [47]. *NDT80* transcription was induced by addition of estradiol in the presence or absence of Mek1-as inhibitor. Inactivation of Mek1 resulted in loss of phosphorylated Hop1 at the 6-hour time point, consistent with repair of DSBs, loss of Hed1 phosphorylation and efficient meiotic progression (Fig 5A and 5B, +1-NA-PP1). Ndt80 was highly phosphorylated, resulting in production of Cdc5 and destruction of Red1 (Fig 5B, +1-NA-PP1). That this high level of phosphorylation occurs only after Mek1 inactivation suggests that Mek1 activity somehow inhibits phosphorylation of Ndt80 by Ime2. In the absence of inhibitor, Ndt80 was inactive. Only a small fraction of cells entered the meiotic divisions (Fig 5A), phospho-Hop1, phospho-Hed1 and Red1 persisted, and Cdc5 was not detected three hours after induction (Fig 5B, -1-NA-PP1). The activation state of Ndt80 at the 6-hour timepoint was therefore determined by whether Mek1 was active (inactive Ndt80) or inhibited (active Ndt80).

Phosphatase treatment of inactive Ndt80 resulted in the loss of the slower migrating species, producing two predominant bands (Fig 5C). The band indicated as “pr-Ndt80” has previously been interpreted to be unphosphorylated Ndt80, while the fastest migrating band (“Ndt80” in Fig 5C) was said to be a “degradation fragment” [53, 63]. Instead, the latter band more likely represents completely unphosphorylated Ndt80 because (1) it runs close to the molecular weight for unmodified Ndt80 (69 kD); (2) the extracts used for these experiments were fixed with trichloroacetic acid prior to lysis and protease inhibitors were included during lysis, making proteolysis unlikely; and (3) this band was not observed when phosphatase inhibitors were included in the reactions (Fig 5C) [53, 63]. We propose that “pr-Ndt80” represents phosphorylated Ndt80 that is more refractile to phosphatase treatment than the phosphorylated forms exhibiting slower mobility. The critical point is that unphosphorylated Ndt80 appeared when inactive Ndt80 was treated with phosphatase, indicating the presence of phosphates.

Determining whether phosphorylation of inactive Ndt80 is dependent upon *MEK1* is difficult because inhibition of Mek1 eliminates the checkpoint by allowing intersister DSB repair [79], resulting in activated Ndt80 and the Ime2-dependent shift [53]. Instead we tested whether phosphorylation of inactive Ndt80 by Ime2 could be ruled out. This goal was accomplished by phosphatase treatment of extracts from a *dmc1Δ NDT80-IN* diploid containing an analog sensitive version of *IME2*, *IME2ΔC241-as*. This allele encodes a truncation of the C-terminus of Ime2 that results in stable, constitutively active kinase [80]. Ime2ΔC241-as activity can be abolished using the 3-MB-PPI inhibitor [48, 81].

Induction of *NDT80* in the *dmc1Δ IME2ΔC241-as* diploid resulted in significantly more meiotic progression than the *dmc1Δ mek1-as NDT80-IN* diploid, although it was much slower than the isogenic *DMC1* diploid, indicating that the checkpoint was active (Fig 5A and 5D). The *IME2ΔC241-as* allele makes hyperactive Ime2 due to the removal of a C-terminal negative regulatory domain [80]. Constitutively high levels of Ime2 activity may be able to counteract the inhibitory phosphorylation of the induced Ndt80 protein better than the endogenous Ime2 activity in the *mek1-as dmc1Δ* diploid, resulting in more progression. Addition of the Ime2ΔC241-as inhibitor decreased the amount of meiotic progression, indicating that the inhibitor was working (Fig 5D). In addition, the slowest migrating Ndt80 species disappeared, Hed1 and Hop1 phosphorylation were stabilized and only a very low level of Cdc5 was detected at the 8-hour time point (Fig 5D and 5E). Therefore, the 7-hour time point in the presence of inhibitor represents inactive Ndt80 that lacks Ime2 phosphorylation. Phosphatase treatment resulted in faster migrating bands, demonstrating that checkpoint inactivated Ndt80 contains Ime2-independent phosphates which we propose are mediated by Mek1 (Fig 5F).

### Constitutive negative charges on the Ndt80 DBD prevent DNA binding *in vitro*

**Fig 6:**
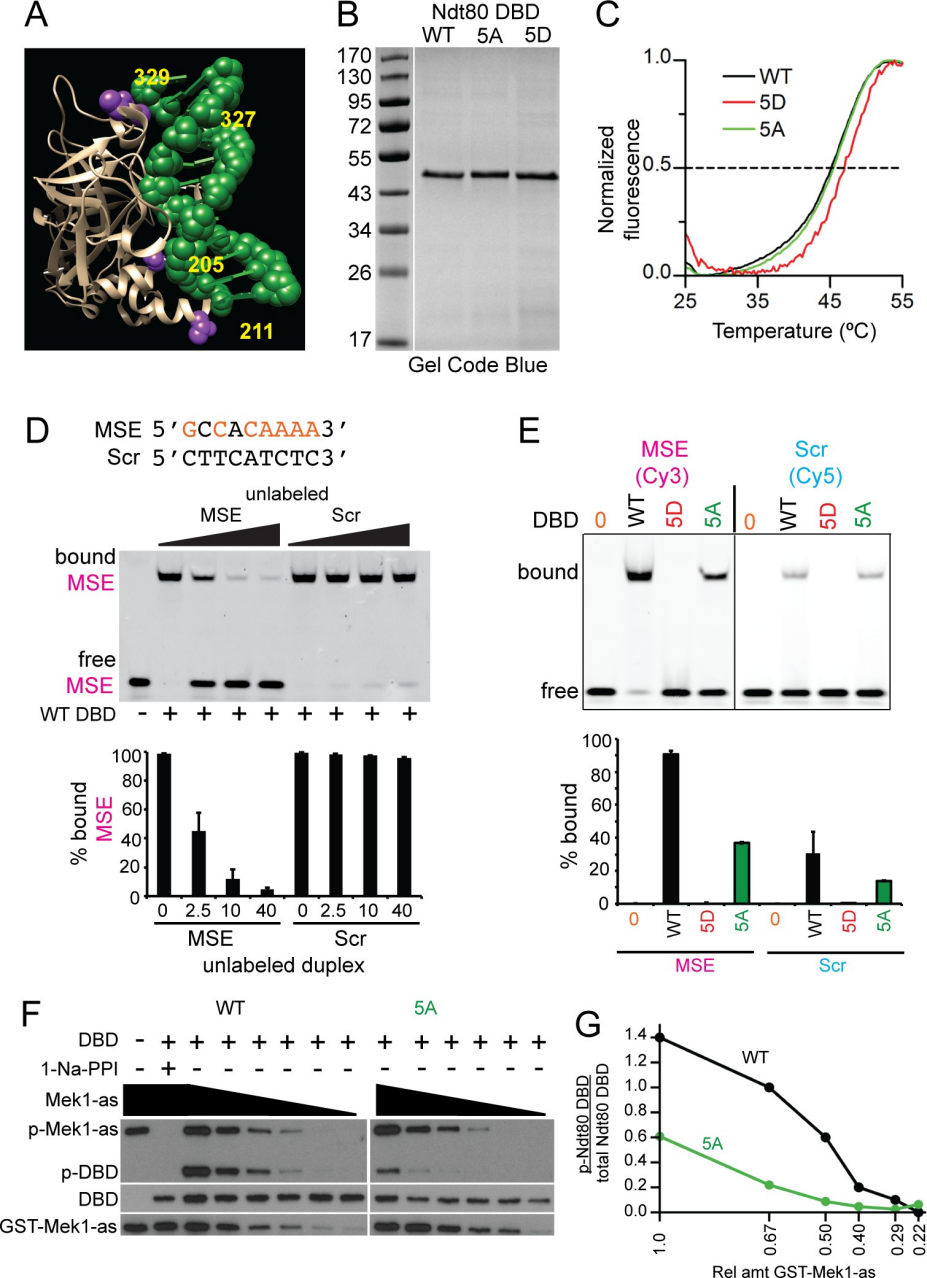
*In vitro* DNA binding and kinase assays using recombinant Ndt80 DBD proteins. (A) Structure of Ndt80 DBD bound to a WT MSE [67]. DBD is tan colored and the MSE is green. Putative Mek1 phosphorylated amino acids (numbered in yellow) are indicated in purple. (B) WT, 5A and 5D DBD proteins after purification from *E*. *coli*. 800 ng protein from the 100 mM imidazole elution for Ndt80 WT, 5A and 5D DBDs (S1 Fig) were fractionated using a 12% SDS-polyacrylamide gel and stained with Gel Code Blue. (C) Melting temperatures (T_m_) were determined by differential scanning fluorimetry (DSF). A representative thermal denaturation curve from one of three independent experiments is shown for each protein. (D) DNA binding assays. Twenty-eight-mer duplexes containing either the nine-base pair MSE from *SPS4* or a non-MSE (Scr) sequence were used. MSE consensus sites are indicated in orange. All reactions contained 10 nM Cy3-labeled MSE duplex (magenta) and 50 nM Ndt80 WT DBD. DNA binding specificity was assessed by the addition of unlabeled MSE or Scr duplexes in increasing concentrations (2.5X, 20X or 40X). Reactions were fractionated on native polyacrylamide gels and Cy3 fluorescence detected using a phosphoimager. Quantification was performed on two independent experiments. Error bars indicate the ranges. (E) Comparison of different DBDs binding to MSE or Scr sequences. The indicated Ndt80 DBD (50 nM) was incubated with either 50 nM Cy3-labeled MSE or Cy5-labeled Scr duplex. Quantification was performed on two replicates run on the same gel for each duplex. Error bars indicate the range. (F) *In vitro* kinase reactions. Reactions contained 40 ng Ndt80 WT or 5A DBD, 100 μM 6-Fu-ATPγS without (-) or with (+) 1 μM 1-Na-PP1. The starting amount of partially purified GST-Mek1-as was 28 ng (= 1) with dilutions of 0.67, 0.50, 0.40, 0.29 and 0.22 as indicated by the black triangles. Thio-phosphorylated proteins were alkylated by the addition of 2.5 mM *p*-nitrobenzylmesylate and the resulting epitopes detected using thiophosphate ester antibodies (p-GST-Mek1-as and p-Ndt80 DBD). Ndt80 DBDs and GST-Mek1-as were detected by probing the same reactions with α-Ndt80 and α-Mek1 antibodies, respectively. (G) Quantification of the kinase assays shown in Panel F. This experiment was conducted three times with similar results.

Crystal structures of the Ndt80 DBD (amino acids 1–340 or 59–330) bound to an MSE show that the Mek1 consensus sites at S205, T211, S327 and S329 are juxtaposed to the sugar-phosphate backbone of the DNA (Fig 6A) [67, 68]. (No structural information is available for S24). Phosphorylation of these sites therefore places negatively charged phosphates in positions where they could repel the negatively charged DNA, thereby preventing DNA binding. This idea was tested using electrophoretic mobility shift assays (EMSA) with recombinant Ndt80 DBD (aa 1–340) and 29-mer duplexes containing either the MSE from *SPS4* or a non-specific sequence designated as Scr (Fig 6D). Note that these DBDs contain the first five Mek1 consensus sites, but not S343. The *SPS4* MSE was previously used for *in vitro* DNA binding assays and structural studies [67].

The Ndt80 WT, 5A and 5D DBDs were fused to a six-histidine tag and purified following the protocol of [82]. All three purified proteins exhibited similar yields and elution profiles (Fig 6B)(S1 Fig). Differential scanning fluorimetry (DSF) was used to determine whether any of the proteins were unfolded. This assay involves incubating proteins with a fluorescent dye and then slowly increasing the temperature to denature the proteins. As the proteins unfold, hydrophobic regions bind the dye, resulting in an increase in fluorescence [83]. The fluorescence values were then normalized to generate melting curves (Fig 6C). Melting temperatures (T_m_) were calculated as temperatures with fluorescence values midway between the two extremes. The Ndt80 WT, 5A and 5D DBDs exhibited similar melting curves with T_m_s of 45.2°C, 45.5°C, and 47°C, respectively. These values indicate that all three proteins were similarly folded, while the 5D protein was even more stable than the WT or 5A protein (Fig 6C).

DNA binding was assayed by incubating Ndt80 WT DBD with a Cy3 fluorescently labeled DNA duplex containing an MSE. All of the duplex was bound, resulting in decreased mobility of the fluorescent DNA (Fig 6D, lane 2). More than 90% of the binding was specific for the MSE sequence, since unlabeled MSE duplex was an effective competitor, decreasing the amount of shifted fluorescent duplex to ~5% (Fig 6D, lanes 3–5). In contrast, equivalent molar amounts of unlabeled Scr duplex did not compete for binding (Fig 6D, lanes 7–9). The Ndt80 5A DBD also bound specifically to the MSE duplex, although less efficiently than WT, while no binding was observed for the 5D protein (Fig 6E, lanes 2–4). Non-specific DNA binding was assessed in a separate reaction using fluorescently labeled Scr duplex. A similar pattern was observed: the WT DBD exhibited the highest level of non-specific binding, followed by the 5A DBD and no binding for the 5D protein (Fig 6E, lanes, 6–8). We conclude that negative charges on the DBD inhibit Ndt80’s ability to interact even non-specifically with DNA.

### GST-Mek1-as directly phosphorylates the Ndt80 DBD *in vitro*

Having recombinant WT and 5A DBDs in hand allowed us to test whether Mek1 directly phosphorylates the Ndt80 DBD *in vitro*. Phosphorylation was detected using the semi-synthetic epitope system [84, 85]. Kinase assays contained active GST-Mek1-as isolated from meiotic yeast cells and the ATP analog, 6-Fufuryl-ATPγS. This ATP analog can fit into the enlarged ATP pocket present in the GST-Mek1-as kinase, but not in the ATP binding pockets of other kinases that may have co-purified with GST-Mek1-as. Phosphorylation by GST-Mek1-as transfers a thio-phosphate onto its substrates which are then chemically alkylated to generate an epitope that is recognized by a commercially available thio-ester antibody. GST-Mek1-as auto-phosphorylation was used as an internal control to show that the kinase reaction worked (Fig 6F, lane 1) [35, 85]. The Ndt80 WT DBD was phosphorylated by GST-Mek1-as (Fig 6F, lane 3). Both GST-Mek1-as autophosphorylation and DBD phosphorylation were eliminated by addition of 1-NA-PP1, confirming that GST-Mek1-as kinase activity was responsible for the signal (Fig 6F, lane 2). The 5A DBD was also phosphorylated by GST-Mek1-as, but less efficiently (Fig 6F, lane 9 and 6G). Decreasing the amount of kinase reduced 5A phosphorylation more rapidly than WT DBD phosphorylation. We conclude that: (1) Mek1 phosphorylates at least one of the Ndt80 DBD consensus sites *in vitro* and (2) Mek1 can also phosphorylate non-consensus sites within the DBD.

## Discussion

### Mek1 phosphorylation of Ndt80 provides a readout for meiotic DSB repair

It has been known for several years that the meiotic recombination checkpoint in yeast requires *MEK1* and that a key target of the checkpoint was Ndt80, but how the two were connected was unclear. The simplest idea, that Mek1 inactivates Ndt80 by directly phosphorylating it, was not considered for two reasons. First, Ndt80 phosphorylation was proposed to promote, not inhibit, Ndt80 activity [48, 63, 64]. Second, deletion of *MEK1* has no effect on the mobility shift of Ndt80, leading to the conclusion that Ndt80 is not a substrate of Mek1 [53]. The latter result is misleading, however, because absence of *MEK1* results in efficient DSB repair using sister chromatids and therefore removes the signal to the checkpoint. As a result, Ndt80 is activated and phosphorylated by Ime2. Therefore, it is impossible to determine whether Ndt80 is phosphorylated by Mek1 under checkpoint arrested conditions simply by comparing Ndt80 mobility shifts in diploids with or without Mek1 activity.

Using a combination of different approaches, we have demonstrated that Mek1 phosphorylation of Ndt80 is responsible for the meiotic recombination checkpoint delay/arrest. First, Ndt80 is phosphorylated when it is inactivated by the meiotic recombination checkpoint and this phosphorylation is independent of *IME2*. Second, Ndt80 contains ten Mek1 consensus phosphorylation sites, eight of which are located either within the DNA binding domain or the “middle region” that is required to inhibit Ndt80 in response to the checkpoint. Preventing phosphorylation at these sites using alanine mutations results in partial bypass of the checkpoint triggered by unrepaired DSBs in the *dmc1Δ* background. Third, Ndt80 contains a conserved five amino acid sequence within the middle region that is required both for checkpoint arrest and for interaction with Mek1. Mutation of this site results in checkpoint bypass without directly affecting Mek1 kinase activity. Fourth, substitution of negatively charged amino acids at Mek1 consensus sites within the Ndt80 DBD constitutively inactivates the transcription factor. Several of these putative Mek1 sites are located immediately adjacent to the negatively charged DNA sugar-phosphate backbone of the MSE. Recombinant Ndt80 DBD containing negative charges at these sites does not bind DNA, even non-specifically. These observations suggest that phosphorylation of the DBD by Mek1 prevents Ndt80 from binding to MSEs and explains how Mek1 phosphorylation can inhibit Ndt80 activity. Finally, Mek1 directly phosphorylates at least one of the Mek1 consensus sites in the Ndt80 DBD *in vitro*, with less efficient phosphorylation of at least one non-consensus amino acid that has not yet been identified.

While disrupting DNA binding may be the major mechanism by which Ndt80 is inactivated by Mek1, it is unlikely to be the only one. Mek1 phosphorylation of Ndt80 at multiple sites appears to inhibit Ndt80 activity in additional ways because preventing phosphorylation of all of the Mek1 consensus sites in the Ndt80 DBD only weakly bypassed the *dmc1Δ* checkpoint arrest. This bypass was increased when the DBD alanine substitutions were combined with alanine mutations within the *bc* domain (*ndt80-10AMS*). Furthermore, the *ndt80-10AMS* checkpoint bypass was less efficient than that observed for the deletion of the RPSKR sequence within the *bc* domain. Since RPSKR is necessary for Mek1 interaction in the two-hybrid system, we propose that deletion of RPSKR disrupts the Mek1-Ndt80 interaction in meiotic cells, preventing any Mek1 phosphorylation of Ndt80 from occurring. In contrast, Mek1 can bind to the Ndt80-10AMS protein via the RPSKR motif and may then phosphorylate non-consensus sites within the DBD and/or the middle region.

Ndt80 that is inactivated by the meiotic recombination checkpoint preferentially localizes to the cytoplasm [69]. It has been proposed that this localization is due to a checkpoint activated cytoplasmic tether but how this tether would work is not clear. An alternative possibility is that Ndt80 constantly shuttles in and out of the nucleus and only when it binds to DNA does Ndt80 remain stably inside the nucleus. Ndt80 is larger than 40 kD, meaning that it is too big to diffuse freely through nuclear pores and must be actively transported [86]. Therefore cytoplasmic localization of inactive Ndt80 could also occur if phosphorylation either promotes nuclear export or inhibits nuclear import. Finally, Mek1 phosphorylation of Ndt80 could inhibit Ime2 phosphorylation at different sites on the transcription factor.

#### Multisite phosphorylation of Ndt80 creates a switch by which meiotic progression is regulated

Multisite phosphorylation provides a mechanism by which biological switches can be generated [87]. For example, in G1, DNA replication is prevented by inhibition of CDK1-Clb activity via the Sic1 protein [88]. Inactivation of Sic1 occurs through protein degradation, which requires phosphorylation of multiple sites that together target Sic1 to the proteasome [87]. A minimum of six sites must be phosphorylated and this occurs by docking CDK-Cln kinases to Sic1 [87, 89]. It takes time to reach the phosphorylation threshold, therefore providing time for the G1 period of the cell cycle [87]. It has been proposed that coordination between DSB repair and meiotic progression occurs via an irreversible switch from low to high CDK levels [54]. We propose that multisite phosphorylation of Ndt80 is the mechanism by which this biological switch is generated.

### Transcriptional and post-transcriptional regulation of *NDT80* coordinates the timing between DSB repair and pachytene exit

**Fig 7:**
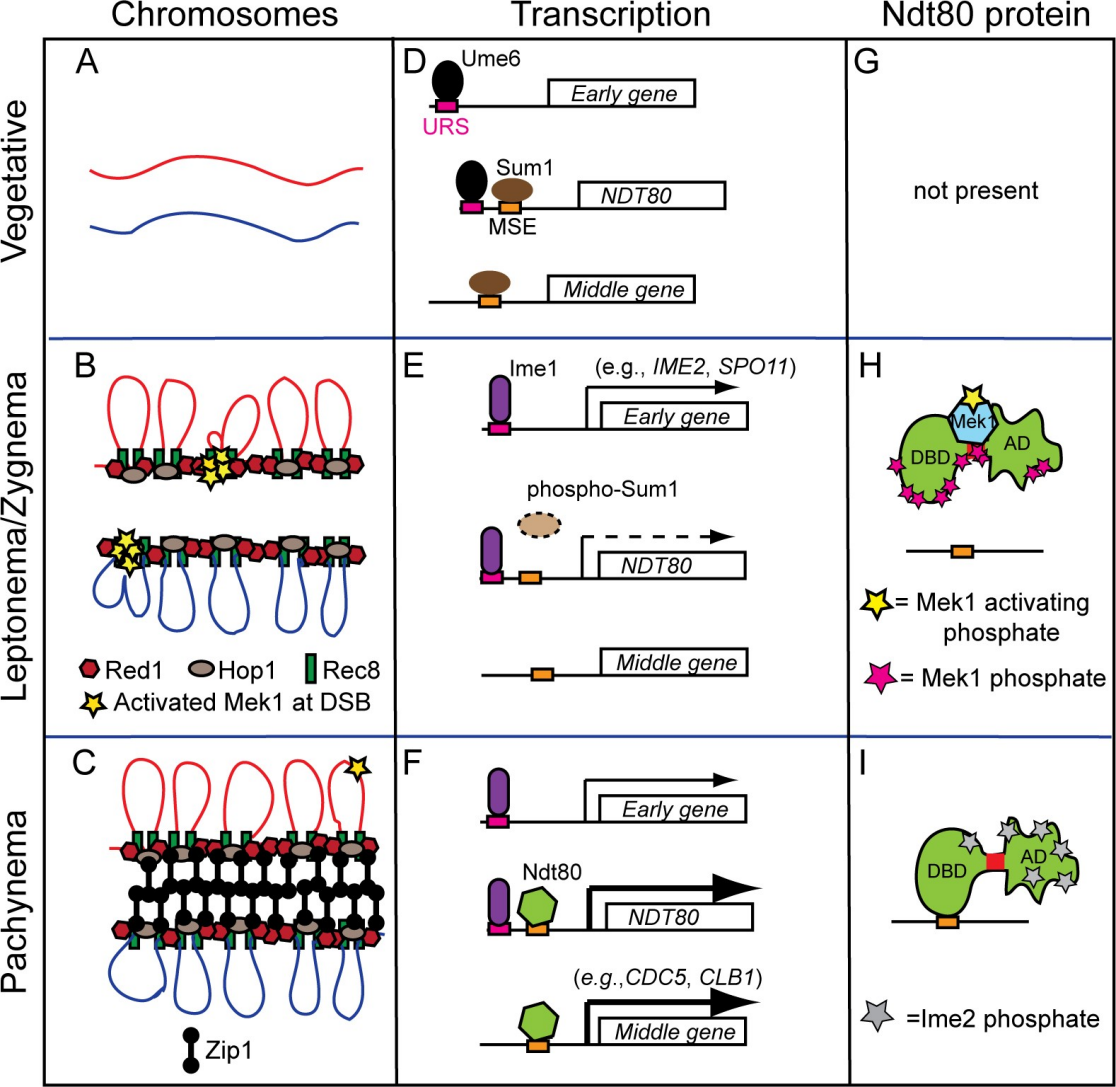
Model for regulation of Ndt80 activity by Mek1. The status of chromosomes, gene transcription and Ndt80 protein at different cell states is shown in three different columns as indicated. (A) Unreplicated pair of homologous chromosomes. (B) Entry into meiosis results in expression of early genes and the condensation of sister chromatids onto axial cores formed of Hop1, Red1 and Rec8 (only one chromatid from each homolog is shown). Hotspot sequences tethered to the axes are cleaved by Spo11, resulting in the recruitment and activation of Mek1 at the axis (yellow stars). (C) Repair of DSBs via the ZMM pathway promotes double Holliday junction formation and synapsis by the insertion of Zip1 between the axial elements to form the SC. Low levels of DSBs continue to occur until the SC is disassembled. (D) In vegetative cells, meiotic early genes and *NDT80* are repressed by the Ume6/Isw2/Sin3-Rpd3 repression complex bound to URS1 sites in their promoters. The Sum1 repressor is bound at MSE sites in the promoters of *NDT80* and middle genes (for simplicity, only one URS1 and one MSE are shown for the *NDT80* promoter). (E) Transfer to sporulation medium results in the replacement of the Ume6 repressor with the Ime1 transcriptional activator at URS1 elements, resulting in early gene transcription (indicated by a solid arrow). Ime1-driven transcription of *NDT80* is delayed (indicated by dashed arrow) until the Ime2 kinase phosphorylates Sum1, thereby allowing its removal from MSEs. (F) Active Ndt80 binds to MSEs in its own promoter to initiate the positive feedback loop, as well as the promoters of middle genes. (G) The *NDT80* gene is not transcribed in vegetative cells so the protein is absent. (H) The low level of Ndt80 protein produced by Ime1 is bound by activated Mek1 and phosphorylated, thereby preventing it from binding to DNA. (I) As chromosomes synapse, the bulk of Mek1 is removed and Mek1 activity decreases. The loss of Mek1 inhibitory phosphorylation, as well as the addition of Ime2 activating phosphates, allows Ndt80 to bind to MSEs and activate transcription.

The following model describes how meiotic gene transcription is integrated with meiotic chromosome structure and DSB repair to promote entry into the meiotic divisions only after DSB repair is complete. In vegetative cells, homologs are not associated and transcription of *NDT80* and its target genes are repressed by Sum1 bound to MSEs [59] (Fig 7A, 7D and 7G). Early meiotic gene expression is prevented by the Ume6 repressor complex bound to a specific Upstream Repression Sequence called URS1 [90, 91] (Fig 7D). Transfer to Spo medium results in the removal of Ume6 and binding of the Ime1 transcriptional activator at URS1 sites, resulting in expression of early genes such as *REC8*, *HOP1*, *RED1*, *MEK1* and *SPO11* [92, 93] (Fig 7E). These gene products (and others) function to assemble AEs, make DSBs and activate Mek1 (Fig 7B). Early in meiosis, when DSBs are first occurring, they are repaired primarily using sister chromatids, indicating that Mek1 activity has not reached the threshold necessary to impose interhomolog bias [94]. Similarly, a threshold amount of Mek1 is necessary to inactivate Ndt80. The cell provides time for Mek1 activation by delaying Ime1-driven *NDT80* transcription through the additional step of removing the Sum1 repressor. This removal requires that Sum1 be phosphorylated by Ime2, along with CDK and DDK (Fig 7E)[57, 60–62]. Since *IME2* is an early gene, it must be transcribed and translated after induction of meiosis. By the time that Ime1-driven transcription of *NDT80* occurs, there is sufficient activated Mek1 (Fig 7B, yellow stars) to phosphorylate Ndt80 (Fig 7H, red stars), thereby preventing Ndt80 from binding to DNA (Fig 7H). Mek1 phosphorylation somehow interferes with Ime2 phosphorylation of Ndt80, which also contributes to keeping the transcription factor inactive. Deletion of *SUM1* from *dmc1Δ* diploids results in bypass of the meiotic recombination checkpoint [72]. We propose that when Ndt80 is prematurely expressed in the *sum1Δ*, there is not enough time to make DSBs and activate Mek1, thereby allowing Ndt80 to activate transcription of its own gene and start the positive feedback loop. A stable interaction between Mek1 and Ndt80 may be necessary to ensure that the kinase is able to counteract removal of phosphates by a phosphatase such as Glc7, which has a role in promoting pachytene exit [95]. As DSBs are processed into double Holliday junctions, their repair promotes chromosome synapsis, resulting in the elimination of most of the Mek1 from chromosomes and a reduction in overall Mek1 kinase activity (Fig 7C) [41, 96]. Without sufficient Mek1 activity, the phosphatase wins out and removes the Mek1-dependent phosphorylation. As a result, Ndt80 becomes activated (which is enhanced by phosphorylation due to Ime2), binds to an MSE in its own promoter to become stably localized to the nucleus and activates a second wave of *NDT80* transcription in a positive feedback loop (Fig 7F and 7I) [48, 57, 63, 64]. In addition, Ndt80 target genes are expressed, including *CDC5* and *CLB1* (Fig 7F). Cdc5 promotes Holliday junction resolution into COs, degradation of Red1 and SC disassembly, thereby eliminating any remaining Mek1 activity [41, 54, 65, 75]. As a result, Rad51 can bind to Rad54 and repair any remaining DSBs prior to entry into MI [41]. Finally, prophase exit resulting from Ndt80-mediated transcription shuts down Spo11 so that no further DSBs are made [97, 98].

### The mechanism of Ndt80 regulation is shared with other Ig-fold transcription factors

While the Ndt80 protein is not conserved outside of fungi, the structure of the DBD is conserved. Ndt80 is a member of the Ig-fold family of transcription factors that includes p53, RUNX, NFAT and NF-kb from mammals [67, 68, 77, 99]. This domain contains a series of loops extending out from several β-sheets that contact DNA to mediate site-specific binding [99]. Interestingly, Ndt80 has more extensive contacts with DNA than other Ig-fold transcription factors, perhaps because Ndt80 binds DNA as a monomer, in contrast to other the proteins which bind as dimers [67, 77].

A common feature of Ig-fold transcription factors is the inhibition of DNA binding by phosphorylation, leading to cytoplasmic localization. For example, NFAT is required for the transcription of cytokine genes involved in T cell activation. In unstimulated cells, phosphorylation of the NFAT nuclear localization signal (NLS) blocks import of the protein into the nucleus [100]. Stimulation of a human T cell lymphoma cell line with phorbol ester activates a phosphatase that removes the phosphorylation at the NLS, allowing translocation into the nucleus where NFAT binds to a specific DNA sequence in its target genes [101]. This binding is inhibited by Cyclosporin A, which results in phosphorylation of the NFAT DBD and cytoplasmic localization of the protein [101]. In another example, phosphorylation of threonine 173 in the DBD of Runx3 by Aurora kinase prevents DNA binding [102]. Similar to the Ndt80 phosphosites, T173 is present at the Runx3-DNA interface. Dissociation from the DNA results in relocalization of Runx3 to the cytoplasm and centrosome during early mitosis. Finally, the p53 protein is a transcription factor that functions in tumor suppression by transcribing genes that promote cell cycle arrest and apoptosis in response to DNA damage [103]. Aurora-A phosphorylates serine 215 in the p53 DBD *in vivo*. A *p53-S215D*, but not *S215A*, mutant prevents DNA binding, resulting in down regulation of target genes necessary for tumor suppression [104]. Although these transcription factors and Ndt80 regulate vastly different processes, there is clearly conservation of the regulatory mechanism that controls them.

### A conserved theme in meiotic recombination checkpoint regulation in yeast and mammals

Many components of the meiotic recombination checkpoint are conserved between yeast and mammals, even though mammalian meiosis is more complicated than yeast, due to the presence of the X and Y sex chromosomes in males and the dictyate arrest that occurs in oocytes after pachytene exit [10]. DSB-dependent checkpoints have been observed in both mouse oocytes and spermatocytes [105–108]. To study the role of the meiotic recombination checkpoint in mice, a hypomorphic allele of the *Trip13* gene called *Trip13*^*mod*^ has been used. This mutant has the advantage that chromosomes synapse but many DSBs remain unrepaired, thereby eliminating signals that might arise from a synapsis checkpoint. *Trip13*^*mod*^ triggers a DSB-dependent arrest in early pachynema that can be distinguished from later arrest points by the absence of a testis-specific histone variant called H1t. Using this assay, the DSB-dependent arrest has been shown to be dependent on *Atm*, *Chk2*, and *HORMAD1/2*, similar to the requirements for the orthologous yeast genes, *TEL1*, *MEK1* and *HOP1*, respectively in the meiotic recombination checkpoint [10]. A key target of the mammalian checkpoint is p53 and its paralog, TAp63. Deletion of *p53* or *TAp63* in *Trip13*^*mod/mod*^ mice allows both oocytes and spermatocytes to progress beyond the early pachytene arrest [105, 106]. Using radiation induced DSBs in oocytes, Bolcun-Filas et al (2104)[105] showed that TAp63 is phosphorylated in a *Chk2*-dependent manner that requires the Chk2 consensus phosphorylation site (LXRXXS) [109]. The mammalian checkpoint response therefore resembles that of yeast: DSBs indirectly activate an FHA-domain containing effector kinase, Chk2 or Mek1, in the context of the AE to regulate an Ig-fold transcription factor, p53/TAp53 or Ndt80, thereby creating an arrest. The main difference is that in mice the checkpoint activates the p53/TAp63 transcription factors while in yeast phosphorylation of Ndt80 prevents transcription.

## Materials and methods

### Plasmids

S1 Table contains a list of plasmids used in this work with the relevant yeast genotypes. S2 Table lists oligonucleotides and their sequences that were used to construct plasmids. Relevant genes in all of the plasmids were sequenced in their entirety by the Stony Brook University DNA Sequencing Facility to ensure that no unexpected mutations were present. Site directed mutagenesis of *NDT80* was carried out using the *URA3 NDT80* integrating plasmid, pHL8 [61] and the protocol in the Quikchange kit (Stratagene) to generate the *2A*, *4AMS*, *5AMS*, *7AMS*, *9AMS*, *10AMS 10DMS*, *6N*, *S24D*, *S343D*, *S205D T211D*, *S327D S329D*, *K374A R375A* and *K374D R375D* mutations. The *ndt80-6A*, *6D* and *8D* alleles, in pNH400, pNH401 and pNH405, respectively, were constructed using three fragment Gibson Assembly (GA) reactions (New England BioLabs). One fragment was pRS306 digested with EcoRI and ClaI [110]. The second fragment was 3.3 kb, with overlapping homology with the EcoRI side of the vector and the *NDT80* gene between codons 379 and 385. It was amplified using the polymerase chain reaction (PCR) with the primers NDT80-WT-EcoRI-F1 and NDT80-R-385. The third fragment was 1.1 kb and contained *NDT80* sequence between codons 379 and 385 and overlapping homology with the ClaI side of the vector. Amplification of this fragment used primers NDT80-WT-ClaI-R1 and NDT80-F-379. For pNH400 and pNH405, pHL8-10AMS was used as the template for the 3.3 kb fragment containing the S24A, S205A, T211A, S327A, S329A and S343A mutations. For pNH401, pHL8-10DMS was the template for the 3.3 kb fragment containing the S24D, S205D, T211D, S327D, S329D and S343D mutations. The 1.1 kb fragment for pNH400 and pNH401 was amplified from the *NDT80* gene in pHL8, while pHL8-2A (T399A T420A) was the template for 1.1kb fragment used to make pNH405. The three fragment GA reaction used to make *NDT80-Δbc* (pHL8-*Δ*bc) used pHL8 to generate two fragments, one using primers NDT80-WT-EcoRI-F1/NDT80-bc-Cla-R1 and the other using NDT80-bc-Cla-F2/NDT80-WT-ClaI-R1, which were then assembled into EcoRI/ClaI digested pRS306.

Estradiol inducible alleles of *NDT80* (*NDT80-IN*) were created using three fragment GA reactions. One fragment was EcoRI/ClaI digested pRS306. The second fragment, containing the *GAL1* promoter with homology on one end to sequences flanking the EcoRI site of pRS306, was amplified using pFA6a-HIS3MX6-P_GAL1_-GFP as the template and P_GAL1_-EcoRI-F1 and P_GAL1_-R1 as primers. The third fragment contained the *NDT80* open reading frame (ORF) and 3’ flanking sequence. One end had homology to the 3’ end of the *GAL1* promoter and the other end to sequences flanking the ClaI site in pRS306. For pBG4, this fragment was amplified using pHL8 as template and the primers, NDT80-ORF-GAL1-F1 and NDT80-WT-ClaI-R1. For pXC11 and pXC12, the template for Fragment 3 was pNH400 and pNH401, respectively.

The *E*. *coli* expression plasmids, pNH407-WT, -5A, and -5D were also constructed using GA. The plasmids contain the *NDT80* DBD (codons 1–340) followed by a stop codon, fused in frame to six histidines in the pET-28a vector (Novagen). 1.1 kb fragments containing the DBD with overlapping homology flanking the Nde1 and XhoI sites of pET-28a were amplified using either pHL8 (WT), pHL8-10AMS (5A) or pHL8-10DMS (5D) and the primers pET28a-NDT80-F and pET28-NDT80-340-R. These fragments were then incubated with pET-28a digested with NdeI and XhoI and the GA reagent.

The *lexA-MEK1* plasmid, pTS3, was constructed using PCR and the primers MEK1-lexA-5/MEK1-lexA-3 to amplify a 1.5 kb fragment containing *MEK1* with BamHI sites engineered onto either end. This fragment was ligated into BamHI-digested pSTT91, resulting in an in-frame fusion of the *MEK1* ORF with *lexA*. The R51A mutation in the FHA domain was introduced into pTS3 by site-directed mutagenesis to make pTS3-R51A [47].

The *GAD-ndt80*^*284-627*^ fusion (plasmid A32) was isolated from a two-hybrid screen using *lexA-MEK1* as bait. This allele was then re-created *de novo* in pXC13 using GA so that direct comparison to various deletion alleles could be made. All *ndt80* sequences were fused in-frame with *GAD* and had the same transcriptional terminator and *NDT80* 3’ untranslated region (UTR). For pXC13, PCR was used to amplify a fragment containing the *GAD-ndt80* fusion from A32 using the primers, NDT80-GAD-F/NDT80-GAD-R. The resulting 1.8 kb fragment was then cloned into pACTII digested with NcoI and XhoI. The K374A R375A and K374D R375D mutations were separately introduced into pXC13 by site-directed mutagenesis to make pXC13-KR>AA and pXC13-KR>DD, respectively. The *GAD-ndt80-Δbc* allele contains an internal, in-frame deletion of the 57 codons of the “bc” domain and was created using a three fragment GA reaction. The first fragment contained *GAD* fused to *NDT80* codons 284–345 and was generated using the primers, NDT80-GAD-F/NDT80-bc-Cla-R1. The second fragment contained *NDT80* codons 403–627 along with overlap with the 3’ end of fragment 1. In this case the primers were NDT80-bc-Cla-F2/NDT80-GAD-R. These fragments were then cloned into NcoI/XhoI digested pACTII to generate pXC14. The pXC18 plasmid contains the 57 amino acid “bc” domain directly fused to *GAD*. Fragment 1 was generated using NDT80-GAD-bc-F3 and NDT80-N1-R1. This fragment was reacted with the *NDT80* 3’UTR fragment and NcoI/Xho1-digested pACTII to make *GAD-bc*. The RPSKR sequence was deleted from *GAD-ndt80* to make *GAD-ndt80-ΔRPSKR* in the following way. Fragment 1 was created using NDT80-GAD-F and NDT80-370-R as primers and pXC13 as template to generate a fragment with homology on one end to the 3’ end of GAD and on the other end to *NDT80* ending at codon 370. The second fragment was amplified using NDT80-RPSKRΔ-F and NDT80-GAD-R with the pXC13 template. This fragment overlaps on one end with *NDT80* codons 360–370, then deletes codons 371–375 and continues to end of *NDT80* and homology to the XhoI digested end of pACTII. These two fragments were joined with NcoI/XhoI-digested pACTII by GA to make pNH318. A similar strategy was used to delete the RPSKR codons from the *NDT80* ORF to make pNH317. The three fragment GA reaction consisted of (1) EcoRI/ClaI digested pRS306; (2) a fragment amplified from pHL8 using NDT80-WT-EcoRI-F1 and NDT80-370-R and (3) a fragment amplified from pHL8 using NDT80-RPSKRΔ-F and NDT80-WT-Cla-RI. To put *GAD-bc* under control of the *MEK1* promoter, a 1.2 kb fragment containing *GAD-bc* was amplifed using YEp-GADbc-F and YEp-GADbc-R with pXC18 as the template. This fragment has one end homologous to the *MEK1* promoter and the other end homologous to sequences downstream of the NdeI site in pDW14. GA was used to introduce the *GAD-bc* fragment into Nde1-digested pDW14 to make pLB1.

### Strains

All strains were derived from the SK1 background unless otherwise noted and their genotypes are listed in S3 Table. Liquid and solid media used for growing cells vegetatively or for sporulation are described in [85]. PCR-based methods were used to delete genes with the drug resistance markers, *kanMX6*, *natMX4* and *hphMX4* [111–113]. All deletions were confirmed by PCR. The presence of the deletion allele was confirmed using a forward primer upstream of the ORF and a reverse primer in the drug resistance gene. The absence of the WT allele was also tested using the same forward primer with a reverse primer internal to the gene’s ORF.

To make NH2081, *NDT80* was deleted from the haploid parents of NH144, which were then mated. The second exon of *DMC1* was then deleted from the *ndt80Δ*::*hphMX4* haploids and mated to make NH2402. The NH144 diploid is heteroallelic for *leu2*, making it impractical to transform with *LEU2* plasmids since transformants cannot be distinguished from mitotic recombinants. The *LEU2* gene was therefore deleted in one of the NH144 parents and then mated to the other to make the *leu2ΔhisG/leu2Δ*::*kanMX6* diploid, NH2444.

The NH2426:pEP105^2^::pX^2^ (where the “2” indicates a homozygous plasmid) series of diploids was constructed by first deleting *NDT80* with *hphMX4* from SKY370 and SKY371. The *TRP1 GAL4-ER* integrating plasmid, pEP105, was digested with Nhe1 and integrated at the *trp1*::*hisG* locus in the resulting haploids [41]. *URA3*-integrating plasmids containing different alleles of *NDT80* were then digested with NsiI to target integration to *ura3*. Integration of the plasmids was confirmed by PCR. The haploids were mated to form homozygous diploids.

The phosphatase experiments were performed NH2437::pEP105^2^::pBG4^2^, which was derived from the haploid parents of NH2092 [114]. These haploids, NH2091-2-4::pJR2 and NH2091-8-2::pJR2 contain pJR2, a *mek1-as URA3* plasmid integrated just upstream of *mek1Δ*::*kanMX6* [114, 115]. Cells that lost the pJR2 plasmid were selected for using 5-fluororotic acid (5-FOA) [116]. To determine whether the *mek1-as* or *mek1Δ*::*kanMX6* allele remained in the chromosome, FOA^R^ colonies were screened for sensitivity to G418. The first 222 bp of the *TRP1* gene were then deleted from the resulting *mek1-as* haploids using *natMX4* [39] and *NDT80* was deleted using *kanMX6*. The *GAL4-ER* fusion was integrated into the 3’end of *TRP1* using NheI-digested pEP105. The *P*_*GAL1*_*-NDT80* plasmid, pBG4, was integrated at the *ura3* locus using NsiI. The haploids were mated to make NH2437::pEP105^2^::pBG4^2^. NH2451 was created by deleting the second exon of *DMC1* with *kanMX6* from the haploid parents of yLJ92 and mating to make the diploid.

### Two-hybrid screen

Two-hybrid experiments were carried out using the L40 strain that contains *lexA* operator sequences upstream of the *HIS3* and *lacZ* genes [66]. *HIS3* expression was assayed on selective medium (SD-leu–trp–his), while *lacZ* was assessed using a colorimetric enzyme assay that produces blue color when ß-galactosidase is present [117]. For the two-hybrid screen, L40 containing pTS3 (2μ *lexA-MEK1 TRP1*) was transformed with a genomic 2μ *LEU2 GAD* fusion library [118] and 1.1 X 10^6^ transformants were screened for growth on SD -leu, -trp, -his medium. Fifteen His^+^ transformants also expressed *lacZ*. The *GAD* plasmids were isolated from the transformants and the fusion junctions sequenced using GAD-AD-5’. One of these transformants contained *GAD* fused in-frame to codons specifying amino acids 284–627 of *NDT80*.

### Spotting assays

Transformants containing different *GAD* plasmids and *lexA-MEK1* were grown overnight at 30°C on a roller in SD-Leu-Trp. The cells were diluted 1:10 in water and the optical density at 660 nm (OD_660_) was determined using a spectrophotometer. Culture volumes equivalent to two ODs were pelleted in microfuge tubes and resuspended in 100 μl sterile water. The cells were transferred to a 96-well plate and ten-fold serial dilutions were made. Ten μl cells were spotted onto SD-Leu-Trp and SD-Leu-Trp-His plates. In addition, four μl of each dilution were plated on a paper filter placed onto an SD-Leu-Trp plate. After growth overnight at 30°C, ß-galactosidase assays were performed. The remaining plates were incubated for three days prior to being photographed.

### Protein purification

Both the 6xHis-Ndt80-WT-DBD (called WT DBD), 6xHis-Ndt80-DBD-5D (called 5D DBD) and 6xHis-Ndt80-DBD-5A (called 5A DBD) proteins were purified from two different 250 ml cell pellets and used for DNA binding assays. Similar results were obtained with both protein preparations. The protein purification protocol was based on the one described in [82].

The *E*. *coli* expression plasmids, pNH407-WT, -5D and 5A were each transformed into BL21(DE3) Codon Plus RIL bacterial cells (Agilent Genomics). Transformants were selected on LB + kanamycin (50 μg/ml) and chloramphenicol (30 μg/ml) (LB +KC) plates. For each plasmid, multiple transformants from a single plate were scraped together and used to inoculate 10 ml LB +KC liquid medium. The cultures were incubated with shaking at 37°C overnight. The next day each culture was diluted to an OD_600_ of 0.02 in 1 L LB + KC in a 4 L flask and the cultures were grown with shaking at 37°C to an OD_600_ of 0.4. Imidazole was added to a final concentration of 1 mM to induce transcription of the tagged *6xHis-ndt80* DBD alleles and the cells remained shaking at 37°C for 4 hours. Each liter of culture was divided into 250 ml aliquots and the cells were pelleted by centrifugation, resuspended in wash buffer (50 mM Tris-HCl, pH 7.5, 100 mM NaCl and 1 mM EDTA), transferred to 50 ml conical tubes and pelleted again. The supernatants were discarded and the cell pellets stored at -20°C.

To lyse the cells, 250 ml cell pellets were thawed on ice and resuspended in 17.5 ml Vershon Lysis Buffer (VLB) (50 mM NaH_2_PO_4_/Na_2_HPO_4_, pH 7.8, 1 M NaCl) containing 5 mM imidazole and 0.2 mM phenylmethylsulfonyl fluoride (PMSF). The imidazole was made fresh and the PMSF added immediately before sonication. Cell suspensions were transferred to pre-chilled 50 mL glass beakers on ice and the cells lysed by sonication using a Qsonica Q500 ultrasonic processor with a 12.7 mm probe (6 pulses of 15 sec, with 30 sec rests) at 70% power. The lysates were then transferred to pre-chilled polyallomer Beckman centrifuge tubes (25 X 80 mm) and centrifuged in a JA-25.50 rotor at 19,647 X g for 30 min at 4°C. The cleared lysates were then transferred to 15 ml conical tubes, flash frozen in liquid nitrogen and stored at -80°C.

To purify the recombinant proteins, the lysates were thawed on ice, distributed between microfuge tubes and spun at 13,000 X g in a microfuge for 10 min to remove any precipitated material. Lysates were pooled and loaded onto a column containing 0.5 ml bed volume of Ni-NTA Superflow agarose beads (Qiagen) equilibrated in VLB+ 5 mM imidazole. Protein bound beads were washed twice with 2.5 ml VLB+ 5 mM imidazole and then subjected to increasing concentrations of imidazole in the following steps: 10 mM, 50 mM, 100 mM, 200 mM and 250 mM. All of the DBDs eluted with the 50 mM and 100 mM steps (S1 Fig). The second 2.5 ml 50 mM imidazole fraction was mixed with the first 2.5 ml 100 mM imidazole fraction and the proteins were concentrated by centrifugation using Amicon Ultra filters (UFC50124). The molar concentrations of the proteins were determined based on the OD_280_ absorbance measured by a NanoDrop spectrophotometer (Thermo Scientific) and the calculated molecular weight of the DBD which is 40,169 g/mole. To visualize the proteins, an appropriate volume of 5 X protein sample buffer was added to each sample and the samples were heated at 95°C for 5 min. Proteins were then fractionated on 12.0% SDS-polyacrylamide gels (1.0 mm spacers), using 250 volts for 25 minutes, and then stained with GelCode Blue (Thermo Scientific).

### Differential scanning fluorimetry (DSF)

DSF was performed on an Applied Biosystems 7500 Fast Real-Time PCR System using the protocol outlined in the “Protein Thermal Shift Studies” User Guide (Applied Biosystems) with minor modifications. DSF experiments used purified Nt80 DBDs at a final concentration of 5 μM in 96-well PCR plates for fast thermocyclers (VWR, Cat. No. 892180296). Each well (50 μL) contained SyproOrange Dye (Sigma Aldrich) diluted to a final concentration of 5x. The plate temperature was ramped from 25°C to 95°C with a linear gradient (1% ramp rate). The fluorescence of the SyproOrange Dye was detected by selecting ROX as the reporter (filter 4, emission range between 600–625 nm). The fluorescence values were normalized to a range between 0.0 and 1.0 using the equation Y_normalized_ = (Y_raw data_−Y_min_)/(Y_max_−Y_min_), where Y_min_ and Y_max_ refer to the minimum and maximum values of fluorescence, respectively. T_m_ values were calculated using the Boltzmann sigmoidal equation in the program GraphPad Prism 4. The Boltzmann sigmoid equation is Y_fluorescence_ = Bottom + (Top—Bottom) / [1 + exp ((V50—X_temp_) / Slope)]. V50 refers to the temperature at which fluorescence is halfway between the bottom and top fluorescence values. T_m_ values are equal to the calculated V50 value.

### Generation of duplexes for electrophoretic mobility shift assays (EMSAs)

Fluorescently labeled 29-mer oligonucleotides (oligos) containing either the *SPS4* MSE sequence (5’ Cy3-ATTGACGCGCG**CCACAAAAA**CGTATCATT) or the Scr sequence (5’ Cy5-ATTGACGCGG**CTTCATCTC**ACGTATCATT)(indicated in bold, respectively) were synthesized by Integrated DNA Technologies with high performance liquid chromatography (HPLC)-grade purity. Unlabeled complementary strands were ordered through the Stony Brook Oligonucleotide Facility. Oligos were resuspended at a concentration of 100 μM in water. Complementary strands were annealed by combining equal amounts of each oligo, adding NaCl to a final concentration of 100 mM, incubating the oligos at 95°C for 5 min and then turning off the hot block to allow the strands to slowly anneal overnight. The resulting Cy3- and Cy5-labeled duplex molecules were purified by size exclusion chromatography using a Superdex 200 Increase 10/300 GL column in eluent A (10 mM Tris-HCl, pH 7.5, 1 mM EDTA, 0.01% NP-40 substitute, 50 mM NaCl) at a flow rate of 0.5 mL/min. The molar concentration of DNA in the peak fractions was quantified based on ultraviolet absorbance at 260 nm using a NanoDrop spectrophotometer.

To visualize the DNA, the appropriate volume of 6 X sucrose buffer (7.2 g sucrose in 10.2 mL 1x TE pH 7.8) was added to each sample, and the DNA was resolved on a 6% polyacrylamide gel (1.5 mm thickness) in 0.5 X TBE at 110V for 45 minutes. In-gel Cy3 or Cy5 fluorescence was detected by a Typhoon 9500 scanner (GE Healthcare). Unlabeled duplexes were constructed similarly, except the Cy3 and Cy5 oligos were replaced with unlabeled oligos and the DNA was visualized by in-gel Sybr Gold staining (Thermo Scientific).

### Electrophoretic mobility shift assays

Protein dilution buffer (20 mM Tris-HCl, pH 8, 50 mM NaCl, 1 mM EDTA, 1 mg/ml bovine serum albumin (BSA), 5 mM 2-mercaptoethanol) and EMSA reaction buffer (10 mM Tris-HCl pH 7.5, 40 mM NaCl, 4 mM MgCl_2_, 6% (w/v) glycerol, 10 mg/ml BSA, 10 μg/ml sonicated salmon sperm DNA) were taken from [82]. DNA binding assays were carried out in 20 μl reactions. Reactions were started by addition of the DBD and were incubated at room temperature for 30 min. Reactions and gels were covered with aluminum foil to minimize exposure of the fluorescently labeled to DNA to light. Competition experiments contained 10 nM (1X) Cy3-MSE and 50 nM WT DBD. Unlabeled MSE or Scr duplex 29-mers were added at 2.5X, 10X or 40X the amount of the labeled duplex. Four μl 6 X sucrose buffer were added to each reaction, which were then immediately loaded onto a 6% 0.5 X TBE gel. The DNA was visualized as described above. Specific and. non-specific DNA binding for the different DBDs were compared using 50 nM of each DBD and 50 nM Cy3-MSE or Cy5-Scr.

### Immunoblots and antibodies

Protein extracts were generated using the tri-chloroacetic acid method described in [119]. A list of primary and secondary antibodies, sources and dilutions can be found in S4 Table.

### Phosphatase experiments

Calf intestinal alkaline phosphatase (AP) treatment of TCA extracts was based on a protocol described in [120] with the following modifications. Sixty μL of extract in 100 mM Tris-HCl, pH 6.8, 4% SDS, 200 mM dithiothreitol (DTT) and 20% glycerol were diluted with 408 μL PMP buffer (50 mM HEPES, pH 7.5, 100 mM NaCL, 2 mM DTT and 0.01% Brij 35). One PhosSTOP tablet (Sigma, Cat. #4906845001) containing phosphatase inhibitors was dissolved in 0.5 ml PMP buffer. For each extract, 4 μL AP (80 units)(Sigma, 11097075001) were added to 40 μL PMP buffer (AP alone), as well as 40 μL PMP buffer plus PhosSTOP inhibitors (AP + Inhibitors) and incubated at room temperature for 30 min. This preincubation step was necessary to get more complete inhibition of the AP. Equal amounts of the diluted extracts (156 μL) were aliquoted into separate microfuge tubes: (1) no AP, (2) AP, and (3) AP plus phosphatase inhibitors. To the “no AP” tube 1, 40 μL PMP and 4 μL AP buffer [25 mM Tris-HCl, pH 7.5, 1 mM MgCl_2_, 0.1 mM ZnCl_2_, 50% glycerol (v/v)] were added; 44 μL AP in PMP buffer was added to tube 2 and 44 μL AP in PMP buffer plus inhibitors was added to tube 3. The final reactions therefore contained 10-fold less protein than the TCA extracts. The reactions were incubated at 30°C for two hours and then stopped by the addition of 5 X Protein sample buffer. The proteins were fractionated on a 7.5% SDS-polyacrylamide gel, transferred to a filter and probed with α-Ndt80 antibodies.

## Supporting information

S1 Data Contains the data and calculations used for all of the numerical data presented in the figures. (XLSX)

S1 Table Plasmids. (PDF)

S2 Table Oligonucleotides. (PDF)

S3 Table Strains. (DOCX)

S4 Table Primary and Secondary antibodies. (PDF)

S1 Fig Protein purification of Ndt80^1-340^ WT, 5A and 5D DBDs. Cleared lysates from cell pellets derived from 250 ml induced bacterial cultures (input) were loaded on Ni-NTA-agarose beads. FT = indicates samples from the flow through. Five ml lysis buffer with the indicated amounts of imidizole were loaded onto the column and collected in two 2.5 ml fractions, labeled “1” and “2”. The asterisk indicates the 100 mM imidizole step that was selected for each protein for use in DNA binding and in vitro kinase assay.
(TIF)
