# Supplementary material for: Mek1 coordinates meiotic progression with DNA break repair by directly phosphorylating and inhibiting the yeast pachytene exit regulator Ndt80
Source: PLoS Genet. 2018 Nov 29;14(11):e1007832. doi: 10.1371/journal.pgen.1007832 (PMC6289461; doi:10.1371/journal.pgen.1007832)
Supplement: S1 Table — (PDF) [file pgen.1007832.s002.pdf]

Table S1. Plasmids

Plasmid name	Relevant yeast genotype	Source
pACTII	2μ *LEU2 GAD*	(Durfee et al., 1993)
pBTM116	2μ *lexA TRP1*	(Hollenberg et al., 1995)
pSTT91	2μ *lexA TRP1 ADE2*	(Woltering et al., 2000)
pRS306	*URA3*	(Sikorski and Hieter, 1989)
pRS316	*CEN ARS1 URA3*	(Sikorski and Hieter, 1989)
pRS316-DMC1	*DMC1 CEN ARS1 URA3*	(Niu et al., 2005)
pLW1	2μ *URA3* P_{MEK1}-*GST-MEK1*	(Wan et al., 2004)
pTS3	2μ *TRP1 ADE2* P_{ADH1}-*lexA-MEK1*	This work
pTS3-R51A	2μ *TRP1 ADE2* P_{ADH1}-*lexA-mek1-R51A*	This work

pXC13	2µ *LEU2* P_{ADH1}*-GAD-ndt80*$^{284-627}$ *=GAD-ndt80*	This work
pXC13-KR>AA	2µ *LEU2* P_{ADH1}*-GAD-ndt80-K374A R375A*	This work
pXC13-KR>DD	2µ *LEU2* P_{ADH1}*-GAD- ndt80-K374D R375D*	This work
pXC14	2µ *LEU2* P_{ADH1}*-GAD-ndt80-Δbc*	This work
pXC18	2µ *LEU2* P_{ADH1}*-GAD-ndt80*$^{346-402}$ *= GAD-bc*	This work
pNH318	2µ *LEU2* P_{ADH1}*-GAD-ndt80-ΔRPSKR*	This work
pDW14	2µ *LEU2* P_{MEK1}	(de los Santos and Hollingsworth, 1999)
pLB1	2µ *LEU2* P_{MEK1}*-GAD-bc*	This work
pHL8	*URA3 NDT80*	(Lo et al., 2012)
pHL8-R177A	*URA3 ndt80-R177A*	(Lo et al., 2012)
pHL8-KR>AA	*URA3 NDT80-K374A R375A*	This work
pHL8-KR>DD	*URA3 ndt80-K374D R375D*	This work
pHL8-Δbc	*URA3 NDT80-Δbc = ndt80*$^{\Delta 346-402}$	This work

pNH317	*URA3 NDT80-ΔRPSKR*	This work
pHL8-2A	*URA3 ndt80-2A = ndt80-T399A T420A*	This work
pHL8-4AMS	*URA3 ndt80-4AMS = ndt80-S386A S391A T399A T420A*	This work
pHL8-5AMS	*URA3 ndt80-5AMS = ndt80-S343A S386A S391A T399A T420A*	This work
pHL8-7AMS	*URA3 ndt80-7AMS = ndt80-S327A S329A S343A S386A S391A T399A T420A*	This work
pHL8-9AMS	*URA3 ndt80-9AMS = ndt80-S205A T211A S327A S 329A S343A S386A S391A T399A T420A*	This work
pHL8-10AMS	*URA3 ndt80-10AMS = ndt80-S24A S205A T211A S327A S329A S343A S386A S391A T399A T420A*	This work
pHL8-10DMS	*URA3 ndt80-10DMS = ndt80-S24D S205D T211D S327D S329D S343D S386D S391D T399D T420D*	This work
pNH400	*URA3 ndt80-6A = ndt80-S24A S205A T211A S327A S329A S343A*	This work
pNH405	*URA3 ndt80-8A = ndt80-S24A S205A T211A S327A S329A S343A T399A T420A*	This work
pNH401	*URA3 ndt80-6D = ndt80-S24D S205D T211D S327D S329D S343D*	This work
pHL8-6N	*URA3 ndt80-6N = ndt80-S24N S205N T211N S327N S329N S343N*	This work
pHL8-S24D	*URA3 ndt80-S24D*	This work
pHL8-S343D	*URA3 ndt80-S343D*	This work
pHL8-S205D T211D	*URA3 ndt80-S205D T211D*	This work
pHL8-S327D S329D	*URA3 ndt80-S327D S329D*	This work

pBG4	*URA3 P_{GAL1}-NDT80*	This work
pXC11	*URA3 P_{GAL1}-ndt80-6A*	This work
pXC12	*URA3 P_{GAL1}-ndt80-6D*	This work
pNH407-WT	*6HIS-ndt80$^{1\text{-}340}$*	This work
pNH407-5A	*6HIS-ndt80$^{1\text{-}340}$-5A = S24A S205A T211A S327A S329A*	This work
pNH407-5D	*6HIS-ndt80$^{1\text{-}340}$-5D = S24D S205D T211D S327D S329D*	This work
pEP105	*P_{GPD}-GAL4.ER TRP1*	(Prugar et al., 2017)
pFA6a-HIS3MX6-PGAL1-GFP	*HIS3MX6-P_{GAL1}-GFP*	(Longtine et al., 1998)
pJR2	*URA3 mek1-as*	(Callender et al., 2016)
pET-28a	*6His kan^R*	Ed Luk (Novagen)

Callender, T.L., Laureau, R., Wan, L., Chen, X., Sandhu, R., Laljee, S., Zhou, S., Suhandynata, R.T., Prugar, E., Gaines, W.A.*, et al.* (2016). Mek1 down regulates Rad51 activity during yeast meiosis by phosphorylation of Hed1. PLoS Genet *12*, e1006226.
de los Santos, T., and Hollingsworth, N.M. (1999). Red1p, a *MEK1*-dependent phosphoprotein that physically interacts with Hop1p during meiosis in yeast. J Biol Chem *274*, 1783-1790.
Durfee, T., Becherer, K., Chen, P.L., Yeh, S.H., Yang, Y., Kilburn, A.E., Lee, W.H., and Elledge, S.J. (1993). The retinoblastoma protein associates with the protein phosphatase type 1 catalytic subunit. Genes Dev *7*, 555-569.
Hollenberg, S.M., Sternglanz, R., Cheng, P.F., and Weintraub, H. (1995). Identification of a new family of tissue-specific basic helix-loop-helix proteins with a two-hybrid system. Mol Cell Biol *15*, 3813-3822.
Lo, H.-C., Kunz, R.C., Marullo, A., Gygi, S.P., and Hollingsworth, N.M. (2012). Cdc7-Dbf4 is a gene-specific regulator of meiotic transcription in yeast. Mol Cell Bio *32*, 541-557.
Longtine, M.S., McKenzie, A., 3rd, Demarini, D.J., Shah, N.G., Wach, A., Brachat, A., Philippsen, P., and Pringle, J.R. (1998). Additional modules for versatile and economical PCR-based gene deletion and modification in *Saccharomyces cerevisiae*. Yeast *14*, 953-961.
Niu, H., Wan, L., Baumgartner, B., Schaefer, D., Loidl, J., and Hollingsworth, N.M. (2005). Partner choice during meiosis is regulated by Hop1-promoted dimerization of Mek1. Mol Biol Cell *16*, 5804-5818.
Prugar, E., Burnett, C., Chen, X., and Hollingsworth, N.M. (2017). Coordination of Double Strand Break Repair and Meiotic Progression in Yeast by a Mek1-Ndt80 Negative Feedback Loop. Genetics *206*, 497-512.
Sikorski, R.S., and Hieter, P. (1989). A system of shuttle vectors and yeast host strains designed for efficient manipulation of DNA in *Saccharomyces cerevisiae*. Genetics *122*, 19-27.
Wan, L., de los Santos, T., Zhang, C., Shokat, K., and Hollingsworth, N.M. (2004). Mek1 kinase activity functions downstream of *RED1* in the regulation of meiotic double strand break repair in budding yeast. Mol Biol Cell *15*, 11-23.
Woltering, D., Baumgartner, B., Bagchi, S., Larkin, B., Loidl, J., de los Santos, T., and Hollingsworth, N.M. (2000). Meiotic segregation, synapsis, and recombination checkpoint functions require physical interaction between the chromosomal proteins Red1p and Hop1p. Mol Cell Biol *20*, 6646-6658.
