# Supplementary material for: Mek1 coordinates meiotic progression with DNA break repair by directly phosphorylating and inhibiting the yeast pachytene exit regulator Ndt80
Source: PLoS Genet. 2018 Nov 29;14(11):e1007832. doi: 10.1371/journal.pgen.1007832 (PMC6289461; doi:10.1371/journal.pgen.1007832)
Supplement: S2 Table — (PDF) [file pgen.1007832.s003.pdf]

Table S2. Oligonucleotides used for plasmid constructions

Name	Sequence
MEK1-lexA-5	5’GAAGAAGAGCGGGATCCGACCGTTGTATAGC3’
MEK1-lexA-3	5’GAAAGAGGAAGAGAGGATCCTATGATTACTC3’
MEK1-R51A-5’	5’CATCAGCTCGTGAAAGTTGGGGCAAATGATAAGGAATGTCAACTC3’
MEK1-R51A’3’	5’GACTTGACATTCCTTATCATTTGCCCCAACTTTCACGAGCTGATG3’
NDT80-GAD-F	5’ACGCTAGCTTGGGTGGTCATATGGCCGATCCAGATACCTTTCATGG3’
NDT80-GAD-R	5’TCAGTATCTACGATTCATAGATCTCGATGTTCCTTATTACAGCAG
NDT80-bc-Cla-R1	5’CCCCCTCGAGGTCGACGGTATCGATAACGTTGCTGTGATAATTAATTC3’
NDT80-bc-Cla-F2	5’GCTTCGAAGGAAATGAGGATC3’
NDT80-GAD-bc-R2	5’TAACGATTTAAAATCATTAGTTTATGACACTCGACGGTGTTCTCAC3’
NDT80-GAD-bc-F3	5’ACGCTAGCTTGGGTGGTCATATGGCCAATTCCTCACAAAACAGC3’
NDT80-N1-R1	5’TAACGATTTAAAATCATTAGTTTATTTATTCCATTGGTGTGGATTG3’
NDT80-WT-EcoRI-F1	5’ TGGATCCCCCGGGCTGCAGGAATTCTAAATAACGGTTTTTACAATGG3’
NDT80-GAD-370-R	5’TGCATTTAAGCAACTTTCATTTAAC3’
NDT80-RPSKRΔ-F	5’GTTAAATGAAAGTTGCTTAAATGCACGATCCAAAGTGGCGCTAGG3’

YEp-GADbc-F	5’ TATACAACAGAAAGAAGAAGAGCCAATGGATAAAGCGGAATTAATTC3’
Yep-GADbc-R	5’GCCTGCAGGTCGACCAACGGTCTCACATACAGTACTGAAGCCG3’
NDT80-R-385	5’TTCGGTGCACCTAGCGCCACTTTGG3’
NDT80-WT-ClaI-R1	5’ CCCCCTCGAGGTCGACGGTATCGATTCCCTTTTGTGAACTTCAAG3’
NDT80-F-379	5’GTGCGCTAGGTGCACCGAACTCTGGGG3’
P_{GAL1}-EcoRI-F1	5’ACTAGTGGATCCCCCGGGCTGCAGGCGGATTAGAAGCCGCCGAG3’
P_{GAL1}-R1	5’CTCCTTGACGTTAAAGTATAG3’
NDT80-ORF-GAL1-F1	5’ACCTCTATACTTTAACGTCAAGGAGCTTAAAATGAATGAAATGG3’
pET28a-NDT80-340R	5’ GATCTCAGTGGTGGTGGTGGTGGTGCTCGAGTTATCTCACAGTTATTCGCT3’
pET28a-NDT80-F	5’GCCTGGTGCCGCGCGGCAGCCATATGAATGAAATGGAAAAC3’
GAD-AD-5’	5’TACCACTACAATGGATG3’
