# Supplementary material for: Mek1 coordinates meiotic progression with DNA break repair by directly phosphorylating and inhibiting the yeast pachytene exit regulator Ndt80
Source: PLoS Genet. 2018 Nov 29;14(11):e1007832. doi: 10.1371/journal.pgen.1007832 (PMC6289461; doi:10.1371/journal.pgen.1007832)
Supplement: S3 Table — (DOCX) [file pgen.1007832.s004.docx]

**Table S1. *Saccharyomyces cerevisiae* strains^a^**

| Name | Genotype | Source |
| --- | --- | --- |
| SKY370 | *MAT***a** *leu2::hisG his4V::LEU2 ura3 lys2 hoΔ::LYS2 arg4-bgl trp1::hisG* | S. Keeney |
| SKY371 | *MATα* *leu2::hisG his4X::LEU2 ura3 lys2 hoΔ::LYS2 arg4-nsp trp1::hisG* | S.Keeney |
| NH144 | *MAT***a** *leu2ΔhisG his4-x ura3 lys2 ho::LYS2 ARG4*  *MATα leu-K HIS4 ura3 lys2 ho::LYS2 arg4-Nsp* | ([Hollingsworth et al., 1995](#_ENREF_3)) |
| NH749 | same as NH144 only *dmc1-2EΔ::natMX4*^b^ *mek1Δ::LEU2* | ([Callender and Hollingsworth, 2010](#_ENREF_1)) |
| NH2081 | same as NH144 only *ndt80Δ::hphMX4* | This work |
| NH2402 | same as NH144 only *dmc1-2EΔ::natMX4* *ndt80Δ::hphMX4* | This work |
| NH2444 | same as NH144 only  *leu2ΔhisG dmc1-2EΔ::natMX4*  *leu2Δ::kanMX6 dmc1-2EΔ::natMX4* | This work |
| NH2426::pEP105^2^ | *MAT***a** *leu2::hisG his4B:LEU2 trp1::hisG::GAL4-ER::TRP1 lys2 ho::LYS2 ura3 arg4-Bgl*  *MATα leu2::hisG his4X:LEU2 trp1::hisG::GAL4-ER::TRP1 lys2 ho::LYS2 ura3 arg4-Nsp*  *ndt80Δ::hphMX4*  *ndt80Δ::hphMX4* | This work |
| NH2426::pEP105^2^::  pHL8^2^ | same as NH2426::pEP105 only *ura3::NDT80::URA3*  *ura3::NDT80::URA3* | This work |
| NH2426::pEP105^2^::  pHL8-R177A^2^ | same as NH2426::pEP105 only *ura3::ndt80-R177A::URA3*  *ura3::ndt80-R177A::URA3* | This work |
| NH2426::pEP105^2^::  pNH400^2^ | same as NH2426::pEP105 only *ura3::ndt80-6A::URA3*  *ura3::ndt80-6A::URA3* | This work |
| NH2426::pEP105^2^::  pNH401^2^ | same as NH2426::pEP105 only *ura3::ndt80-6D::URA3*  *ura3::ndt80-6D::URA3* | This work |
| NH2426::pEP105^2^::  pBG4^2^ | same as NH2426::pEP105 only *ura3::P_GAL1_-NDT80::URA3*  *ura3::P_GAL1_-NDT80::URA3* | This work |
| NH2426::pEP105^2^::  pXC11^2^ | same as NH2426::pEP105 only *ura3::P_GAL1_-ndt80-6A::URA3*  *ura3::P_GAL1_-ndt80-6A::URA3* | This work |
| NH2426::pEP105^2^::  pXC12^2^ | same as NH2426::pEP105 only *ura3::P_GAL1_-ndt80-6D::URA3*  *ura3::P_GAL1_-ndt80-6D::URA3* | This work |
| NH2437::pEP105^2^::pBG4^2^ | *MAT***a** *leu2 arg4-Nsp ho::LYS2 lys2 ura3::P_GAL1_-NDT80::URA3 dmc1Δ::LEU2 mek1-as*  *MATα leu2 arg4-Nsp ho::LYS2 lys2 ura3::P_GAL1_-NDT80::URA3 dmc1Δ::LEU2 mek1-as*  *lys4Δ::hphMX4 trp1-5’Δ::natMX4::GAL4-ER::TRP1 ndt80Δ::kanMX6*  *lys4Δ::hphMX4 trp1-5’Δ::natMX4::GAL4-ER::TRP1 ndt80Δ::kanMX6* | This work |
| L40^c^ | *MAT***a** *his3Δ200 trp1-90 leu2-3,112 ade2 lys2::lexA_op_-HIS3::LYS2 gal80 ura3:: lexA_op_-lacZ::URA3* | ([Hollenberg et al., 1995](#_ENREF_2)) |
| yJL92 | *MAT***a** *leu2::hisG ho::LYS2 his3::hisG trp1::hisG P_GAL1_-NDT80-::TRP1*  *MATα leu2::hisG ho::LYS2 his3::hisG trp1::hisG P_GAL1_-NDT80-::TRP1*  *ura3::P_GPD1_-GAL4(848).ER::URA3 IME2∆C241-as dmc1Δ::kanMX6*  *ura3::P_GPD1_-GAL4(848).ER::URA3 IME2∆C241-as dmc1Δ::kanMX6* | ([Jin et al., 2015](#_ENREF_4)) |
| NH2451 | Same as yJL92 except *dmc1Δ::kanMX6* | This work |

^a^ All strains were derived from the SK1 background unless otherwise noted.

^b^The *dmc1-2E∆* nomenclature indicates a deletion of the second exon of *DMC1*.

^c^This strain is from the SEY6210/SEY6211 background ([Robinson et al., 1988](#_ENREF_5)).

Callender, T.L., and Hollingsworth, N.M. (2010). Mek1 suppression of meiotic double-strand break repair is specific to sister chromatids, chromosome autonomous and independent of Rec8 cohesin complexes. Genetics *185*, 771-782.

Hollenberg, S.M., Sternglanz, R., Cheng, P.F., and Weintraub, H. (1995). Identification of a new family of tissue-specific basic helix-loop-helix proteins with a two-hybrid system. Mol Cell Biol *15*, 3813-3822.

Hollingsworth, N.M., Ponte, L., and Halsey, C. (1995). *MSH5*, a novel MutS homolog, facilitates meiotic reciprocal recombination between homologs in *Saccharomyces cerevisiae* but not mismatch repair. Genes Dev *9*, 1728-1739.

Jin, L., Zhang, K., Xu, Y., Sternglanz, R., and Neiman, A.M. (2015). Sequestration of mRNAs modulates the timing of translation during meiosis in budding yeast. Mol Cell Biol *35*, 3448-3458.

Robinson, J.S., Klionsky, D.J., Banta, L.M., and Emr, S.D. (1988). Protein sorting in Saccharomyces cerevisiae: isolation of mutants defective in the delivery and processing of multiple vacuolar hydrolases. Mol Cell Biol *8*, 4936-4948.
