# Supplementary material for: Mek1 coordinates meiotic progression with DNA break repair by directly phosphorylating and inhibiting the yeast pachytene exit regulator Ndt80
Source: PLoS Genet. 2018 Nov 29;14(11):e1007832. doi: 10.1371/journal.pgen.1007832 (PMC6289461; doi:10.1371/journal.pgen.1007832)
Supplement: S4 Table — (PDF) [file pgen.1007832.s005.pdf]

**Table S4. Primary and Secondary antibodies.**

| Protein | Primary | dilution | source | Secondary | Dilution | Source |
|---|---|---|---|---|---|---|
| Arp7 | α-Arp7 | 1:50,000 | Santa Cruz sc-8961 | α-Goat | 1:15,000 | Santa Cruz sc-2354 |
| Cdc5 | α-Cdc5 (yC-19) | 1:500 | Santa Cruz sc-6733 | α-Goat | 1:15,000 | Santa Cruz sc-2354 |
| Clb1 | α-Clb1 | 1:300 | Santa Cruz sc-7647 | α-Goat | 1:10,000 | Santa Cruz sc-2354 |
| Hed1 | α-Hed1 | 1:50,000 | Patrick Sung | α-Rabbit | 1:5,000 | Santa Cruz sc-2357 |
| Hed1 p-T40 | α-pT40 | 1:20,000 | N. M. Hollingsworth | α-Rabbit | 1:5,000 | Santa Cruz sc-2357 |
| Hop1 | α-Hop1 | 1:10,000 | N. M Hollingsworth | α-Rabbit | 1:5,000 | Santa Cruz sc-2357 |
| Red1 | α-GST-Red1 | 1:10,000 | N. M Hollingsworth | α-Rabbit | 1:5,000 | Santa Cruz sc-2357 |
| Zip1 | α-Zip1 (y300) | 1:3,000 | Santa Cruz sc-33733 | α-Rabbit | 1:50,000 | Santa Cruz sc-2004 |
| HA | α-HA (12CA5) | 1:2000 | BABCO | α-Mouse | 1:10,000 | Santa Cruz sc-2005 |
| Mek1 | α-Mek1 | 1:20,000 | N. M. Hollingsworth | α-Guinea Pig | 1:10,000 | Santa Cruz sc-2903 |
| Rec8 | α-Mek1 | 1:100,000 | N. M. Hollingsworth | α-Guinea Pig | 1:10,000 | Santa Cruz sc-2903 |
| Ndt80 | α-Ndt80 | 1:15,000 | Michael Lichten | α-Rabbit | 1:5,000 | Santa Cruz sc-2357 |
| Rad54 | α-Rad54 | 1:5,000 | Wolf Heyer | α-Rabbit | 1:5,000 | Santa Cruz sc-2004 |

| | | | | | | |
|---|---|---|---|---|---|---|
| GAD | $\alpha$-GAD | 1:2500 | Takara | $\alpha$-Mouse | 1:10000 | Santa Cruz sc-2005 |
