# Supplementary figures and images for: Mek1 coordinates meiotic progression with DNA break repair by directly phosphorylating and inhibiting the yeast pachytene exit regulator Ndt80
Source: PLoS Genet. 2018 Nov 29;14(11):e1007832. doi: 10.1371/journal.pgen.1007832 (PMC6289461; doi:10.1371/journal.pgen.1007832)

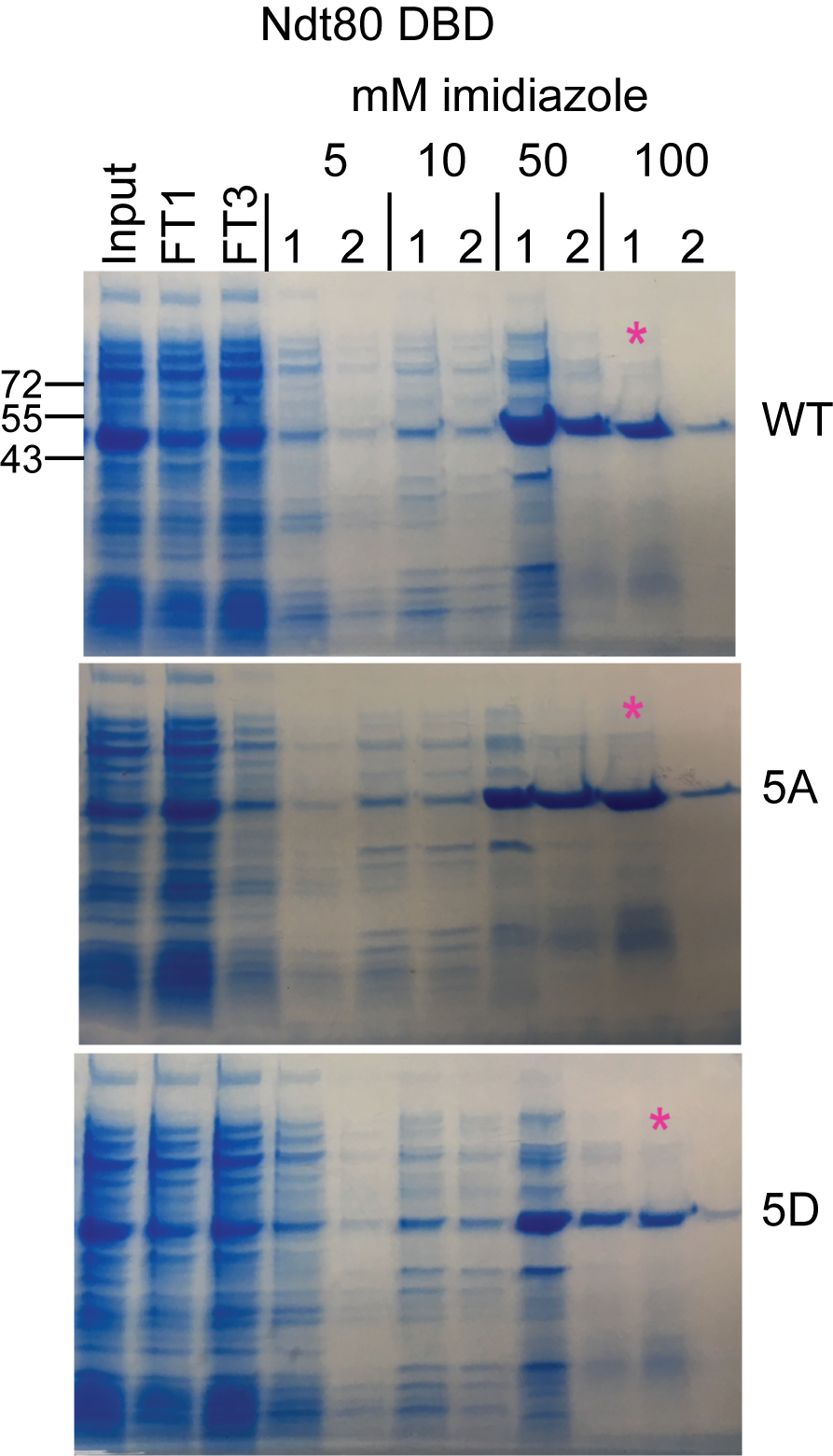

Supplement: S1 Fig — Cleared lysates from cell pellets derived from 250 ml induced bacterial cultures (input) were loaded on Ni-NTA-agarose beads. FT = indicates samples from the flow through. Five ml lysis buffer with the indicated amounts of imidizole were loaded onto the column and collected in two 2.5 ml fractions, labeled “1” and “2”. The asterisk indicates the 100 mM imidizole step that was selected for each protein for use in DNA binding and in vitro kinase assay. (TIF) [file pgen.1007832.s006.tif]
